# Polyethyleneimine (PEI) molecular weight alters the photophysical and FRET properties of water dispersible NaYF_4_:Yb,Er upconversion nanoparticles

**DOI:** 10.1039/d6nr02308h

**Published:** 2026-11-04

**Authors:** Rebecca McGonigle, Christian Homann, Ciera Connelly, Marta Gajewska, Niko Hildebrandt, Robert Pal, Shiao Chow, Ute Resch-Genger, Lewis E. MacKenzie

**Affiliations:** a Department of Pure and Applied Chemistry, University of Strathclyde, Technology and Innovation Centre 99 George Street Glasgow G1 1RD UK l.mackenzie@strath.ac.uk; b Department of Pure and Applied Chemistry, University of Strathclyde, Thomas Graham Building G1 1XL Glasgow UK; c Department of Analytical Chemistry (Dpt.1), Federal Institute for Materials Research and Testing (BAM), Zweiggelände Adlershof Richard-Willstätter-Straße 11 12489 Berlin Germany; d Academic Centre for Materials and Nanotechnology, AGH University of Krakow Mickiewicza 30 30-059 Krakow Poland; e McMaster University, Department of Engineering Physics Hamilton Ontario M8S 4K1 Canada; f Department of Chemistry, Durham University South Road Durham DH1 3LE UK

## Abstract

Upconversion nanoparticles (UCNPs) are optically active materials with near-infrared excitation and tuneable anti-Stokes emission, typically resulting in visible emission. A route to routine and reliable synthesis of water-dispersible UCNPs for biomedical imaging applications is the polyethyleneimine (PEI)-assisted solvothermal autoclave synthesis; however, the properties of the resulting UCNPs remain understudied. Herein, we report how PEI molecular weight used in synthesis alters the properties of core-only, red-emitting, water-dispersible PEI-UCNPs. In terms of brightness, 1.2 kDa PEI-UCNPs exhibited 5 times more intense upconversion luminescence (UCL) than 25 kDa PEI-UCNPs and a 29% longer UCL lifetime (LT) for the red emission band (∼640–690 nm, ^4^F_9/2_ → ^4^I_15/2_ Er^3+^ transition) in DI H_2_O. Dispersal in D_2_O increased emission across wavebands, with green emission (^2^H_11/2_, ^4^S_3/2_ → ^4^I_15/2_ Er^3+^ transition) increasing by 248–365%, whereas red emission only increased by 38–64%. From our results, we infer that PEI may potentially quench the UCL *via* high-energy NH vibrations. We also examined Förster Resonance Energy Transfer (FRET) between PEI-UCNPs and an electrostatically adsorbed fluorescent model dye, AlexaFluor647® (AF647). 25 kDa PEI-UCNPs achieved highest LT-estimated FRET efficiencies of ∼11% in H_2_O and 16% in D_2_O, respectively. These findings highlight that polymer MW should be considered when optimising UCNP synthesis.

## Abstract 2

PEI molecular weight influences the morphology, upconversion luminescence and FRET response of NaYF_4_:Yb,Er nanoparticles, highlighting the importance of polymer selection in UCNP design.

## Graphical abstract

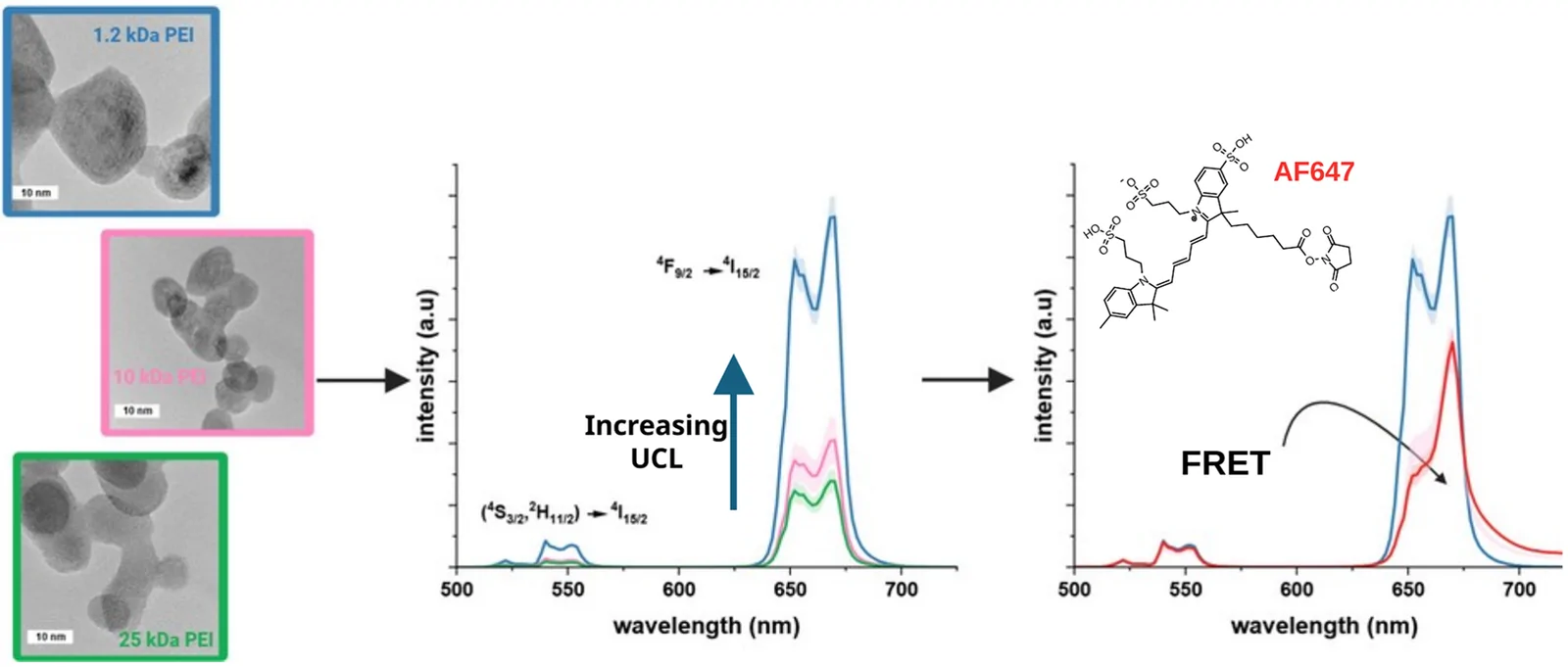

## 1. Introduction

Upconversion nanoparticles (UCNPs) have gained significant interest in bioimaging, due to their near-infrared (NIR) excitation (typically 980 nm), providing a relatively high penetration depth of tissue with no visible autofluorescence compared to visible excitation wavelengths.^1^ UCNPs typically exhibit multi-band visible emission, long upconversion luminescence lifetime decays (LTs) [*i.e. τ* ∼ 100 µs], and do not photobleach.^2–4^ In comparison, organic dyes and most quantum dots typically exhibit short emission LTs [*τ* ∼ ns], may photo bleach/blink, and are often excited at high-energy wavelengths that offer poor tissue depth penetration.^5,6^ Upconversion luminescence (UCL) is facilitated in UCNPs *via* multiple lanthanide (Ln^3+^) ions such as Yb^3+^, Er^3+^, Nd^3+^, and Tm^3+^ arrayed within the low phonon-energy host lattice (*e.g.* NaYF_4_, NaGdF_4_, LiFY_4_, *etc.*).^7,8^ Sensitiser Ln^3+^ ions such as Yb^3+^ can generate UCL by successive absorption of multiple low-energy (NIR) photons through ladder-like energy levels,^9^ with non-radiative energy transfer to emissive Ln^3+^ ions such as Er^3+^ (see Fig. 1). Additionally, UCNPs can serve as magnetic resonance imaging (MRI) contrast agents *via* incorporation of Gd^3+^ or Mn^2+^.^8,10^ These properties can make UCNPs advantageous for biomedical imaging, particularly red-emitting UCNPs, which can reportedly be detected *via* diffuse optical imaging *via* ∼1 cm of overlying tissue.^11–13^

**Fig. 1:**
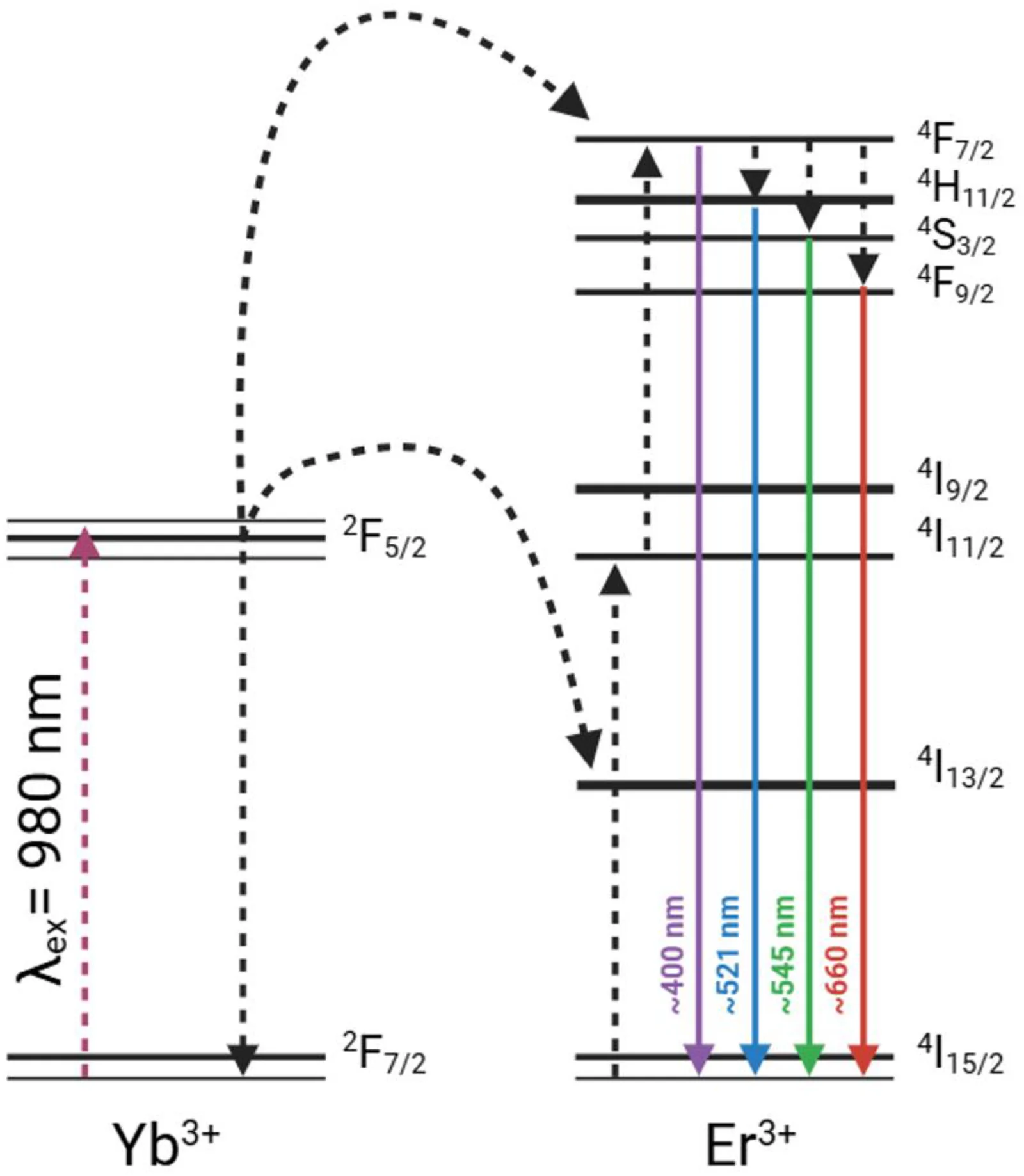
Simplified energy level diagram depicting the upconversion process in NaYF_4_:Yb,Er UCNPs where Yb^3+^ are the sensitiser ions and Er^3+^ the emitter ions.^14^

Surface modification of UCNPs can affect the efficiency of this upconversion process, specifically in aqueous environments; water molecules exhibit high-energy vibrational modes and specifically the OH-stretching vibrations (3300–3700 cm^−1^) can couple with the energy levels of lanthanide ions in UCNPs, resulting in the quenching of UCL.^15^ While UCNPs typically exhibit the brightest emission in organic solvents, water-dispersible particles are preferred for biological applications due to improved biocompatibility and cellular uptake.^16–19^ Therefore, surface modification with water-dispersible polymers or other organic compounds, like polyvinylpyrrolidone (PVP),^10,14^ polyacrylic acid (PAA),^20^ polyethyleneimine (PEI), polydopamine, or gum arabic (GA),^20–22^ are desirable for biomedical applications.^23^

A reliable and straightforward route for the synthesis of water-dispersible UCNPs is the PEI-assisted method, first reported by Wang *et al.*, in 2006,^24^ and subsequently adapted in other studies (see Table 1).^24–27^ A generalised reaction scheme is shown in Fig. 2a. PEI presents positively charged, protonated amine groups, which enable attachment *via* electrostatic binding. There are two forms of PEI, branched and linear (see Fig. 2b), with branched being more commonly available *via* commercial vendors. It is worth noting that linear PEI is not considered in this work, and any reference to PEI herein refers to branched PEI. PEI-UCNPs have been reported for applications including intracellular pH sensors, the detection of antibiotic residues in food and environmental samples, and as multifunctional platforms to image and trigger the release of pro-drugs in cancer treatment therapy.^28–30^ Notably, several studies have used PEI-UCNPs for Förster Resonance Energy Transfer (FRET)-based applications (see Table 1).^25–27^ FRET is a non-radiative energy transfer process between a donor (*e.g.*, emissive lanthanide ions from a UCNP) and an acceptor (*e.g.*, a fluorescent dye), which is extremely sensitive to the distance between the donor and the acceptor. UCNP-based FRET sensors are attractive for quantifying biological interactions and molecular diagnostics *via* spectroscopy or microscopy.^31^ For biosensing and imaging applications, it is necessary to ensure that UCNP surface modifications permit both water dispersibility, promote bright UCL, and maximise FRET efficiency.

**Fig. 2:**
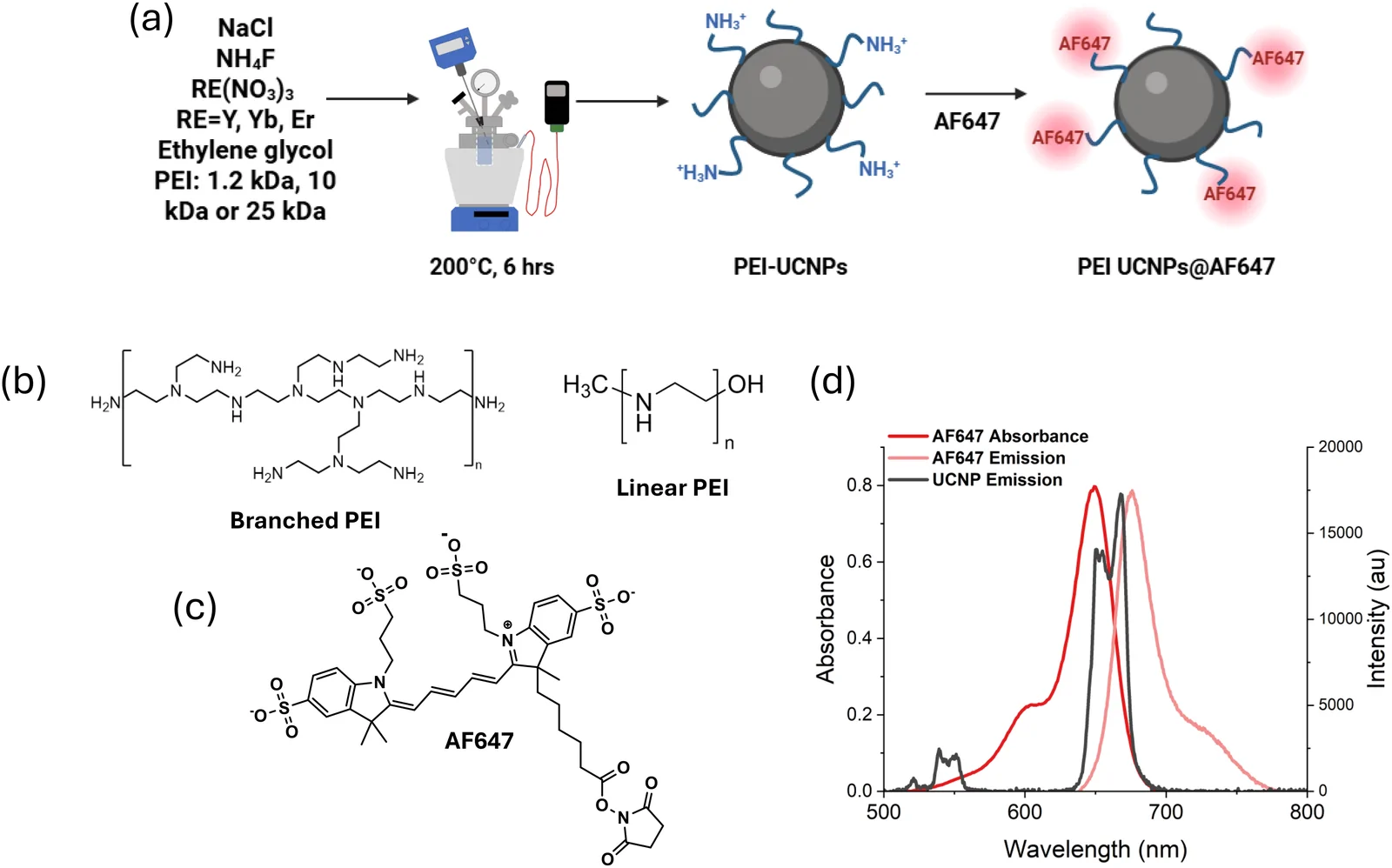
The one-pot solvothermal synthesis of PEI-UCNPs and subsequent loading with AF647. (a) General reaction scheme; (b) structure of branched and linear PEI; the amine (NH_2_) groups on PEI enable AF647 loading. (c) Structure of fluorescent dye AF647. (d) Optical absorption and fluorescence emission of AF647 (5 μM) in H_2_O.

**Table 1:** Summary of literature reports of PEI-UCNPs as FRET donors

Study	UCNP synthesis method	PEI MW used	FRET donor	FRET acceptor	Estimated FRET efficiency	Application
Ding *et al.* (2019)^27^	*PEI in situ*, solvothermal^a^	MW 25 000 kDa	NaGdF_4_:Yb,Er UCNPs	GM-Ag_2_S NDs^b^	57% – LT based estimate	*In vivo* pH sensing in zebrafish
Cui *et al.* (2015)^26^	*PEI in situ*, solvothermal^a^	Not stated	NaYF_4_:Yb,Er UCNPs	CdTe QDs^c^	28% – LT based estimate	Sensing Hg^2+^ in blood serum
Xu *et al.* (2014)^25^	*PEI in situ*, solvothermal^a^	Not stated	NaYF_4_:Yb,Tm UCNPs	TGA capped CdTe QDs^c^	21% – LT based estimate	Sensing Pb^2+^ in blood serum

a Similar to synthesis detailed herein.

b Nanodots.

c Quantum dots.

An unexplored parameter for such systems is PEI molecular weight (MW), which is a proxy for polymer chain length. PEI MW is typically reported as the manufacturer's stated average MW with no quantitative insight into the actual MW distribution of the PEI used in UCNP synthesis. In addition, the PEI MW used is sometimes not even stated in UCNP studies (see Table 1). We hypothesised that varying PEI MW may influence quenching interactions between PEI-UCNPs and their aqueous environment, as well as mediate FRET properties, parameters crucial for applications in biosensing and bioimaging, with model dye molecule AF647, see Fig. 2(c) and (d). The aim of this study was to test how PEI MW alters PEI-UCNP UCL intensity, LT, and FRET efficiency.

## 2. Experimental

### 2.1. Materials

The following reagents were procured from Sigma Aldrich: NaCl (>99.5%), branched polyethyleneimine (PEI) [manufacturer stated molecular weight 1.2 kDa, 10 kDa, and 25 kDa], hydrated rare earth nitrates RE(NO_3_)_3_ (RE = Y, Yb, Er, 99.99%), ethylene glycol (>99%), NH_4_F (>99.99%), AlexaFluor647® NHS ester (AF647), dimethyl sulfoxide (DMSO) (>99.9%), deuterium oxide (D_2_O) (99.9%), deionised (DI) water (RS PRO). All materials were used without further purification.

### 2.2. Methods

#### 2.2.1. PEI-UCNP synthesis *via* solvothermal autoclave reaction

NaCl (2.5 mmol), Y(NO_3_)_3_·6H_2_O (0.78 mmol), Yb(NO_3_)_3_·5H_2_O (0.2 mmol), Er(NO_3_)_3_·5H_2_O (0.02 mmol) and branched PEI (400 mg) [of various stated MW] were introduced to 15 mL of ethylene glycol and magnetically stirred (800 RPM, 50 °C) for 30 min, resulting in a transparent solution (Solution A). Then NH_4_F (4 mmol) was dissolved in 10 mL of ethylene glycol (Solution B). Solution B was then added to Solution A (total volume 25 mL) and magnetically stirred for a further 15 min.

A high-safety bench-top autoclave reactor (PressureSyn®, Asynt Ltd), was used for the PEI-UCNP synthesis (see Fig. 2a). This autoclave reactor had a maximum operating temperature and pressure of 200 °C and 200 barg, pressure indicator, pressure-release valve, and over-pressure emergency release valve.^32^ The reaction mixture was situated in a 50 mL volume glass liner, and a magnetic stirrer bar was added. To reduce (but not eliminate) oxygen in the reaction, N_2_ was introduced to the glass liner for 1 minute prior to the introduction of the reaction solution. After addition, another flow of N_2_ gas was introduced for a further minute to fill the headspace of the autoclave reactor with N_2_. The autoclave reactor was then sealed and heated *via* a hotplate stirrer (IKA® RCT basic) and temperature control *via* a thermocouple (IKA® ETS-D5) situated within the interior of the autoclave reactor and in contact with the reaction mixture. Once an internal reactor temperature of ∼200 °C was reached, the reaction continued for 6 h under constant magnetic stirring at 800 RPM. The pressure in the reactor was ∼1 bar for the duration of the reaction, indicating no significant elevated pressure. This reaction resulted in an orange/yellow solution containing PEI-UCNPs. The resulting PEI-UCNPs were collected and washed 3 times with DI water *via* centrifugation at 17 000*g* for 30 min. The PEI-UCNPs were finally redispersed in 5 mL DI water, forming a cloudy white solution.

Following the reaction, the autoclave and glass liner were washed with soapy water, rinsed with DI water and finally rinsed with acetone, ensuring both were completely dry. To test reproducibility of this synthetic procedure, PEI-UCNP synthesis was undertaken in triplicate for each PEI MW.

#### 2.2.2. ICP-MS of PEI-UCNPs

Dry powder PEI-UCNP samples were prepared *via* evaporation in glass vials, and 3 mg were placed in 2 mL Eppendorf tubes (in triplicate). 1 mL of ultra-pure water (resistivity of 18.2 MΩ) was added to each sample, then the sample was transferred into a perfluoroalkoxy alkane (PFA) microwave digestion tube (MARS Xpress 75 mL) *via* a pipette. To remove any possible remains of the sample from the sample tube, the procedure was repeated with 1 mL concentrated hydrochloric acid (HCl, 37%, Trace Metal Grade, Fisher Chemical); then with 1 mL concentrated nitric acid (HNO_3_, 67%, Trace Metal Grade, Fisher Chemical). An additional 1 mL of HCl and 3 mL of HNO_3_ were then directly added into the microwave digestion tube. Samples were acid digested using a Microwave Assisted Reaction System (MAE, MARS-5, CEM Corporation), following the microwave operating parameters: operating power 800 W; ramp for 20 min up to 200 °C; hold time 20 min at 200 °C. Sample digests were brought up to 50 mL in volumetric flasks using ultra-pure water (18.2 MΩ resistivity) and then filtered through a 0.45 μm cellulose acetate syringe filter (Millex).

#### 2.2.3. Molecular loading of AF647

10 mg mL^−1^ of PEI-UCNPs (of each PEI MW) and AF647 (5 and 10 μM in DMSO) were combined by stirring (30 min), and the resultant solution (made up to 1 mL) was centrifuged at 10 000*g* for 10 min. The supernatant containing excess AF647 was removed. AF647-loaded PEI-UCNPs are referred to herein as PEI-UCNPs@AF647. The resultant pellet of PEI-UCNP@AF647 was resuspended *via* vortexing in DI water (1 mL) for further analysis, and the molecular loading percentage was determined as detailed in section 2.2.7, using UV-Vis spectroscopy (see SI Fig. S1).

#### 2.2.4. Dispersion in D_2_O

10 mg mL^−1^ of PEI-UCNPs (of each PEI MW) in DI water were centrifuged at 17 000*g* for 20 min. The resultant pellet was redispersed in 1 mL of D_2_O using sonication.

#### 2.2.5. UCL LT measurements, and estimations of FRET efficiency *via* LT and total intensity

UCL emission spectra and UCL LT measurements were acquired using an upconversion spectrometer (FS5 V2, Edinburgh Instruments Ltd). Samples (1 mL volume, 10 mg mL^−1^) were contained in 1 cm pathlength quartz cuvettes (Hellma GMBH) in a temperature-controlled cuvette holder set to 20 °C for all measurements. Excitation was provided by a 980 nm laser (2 W max power output) controlled *via* a variable power control unit (MDL-III-980-2W, Edinburgh Instruments Ltd). Steady-state UCL were recorded with an excitation power of 0.56 W with a rectangular illumination spot size of 2 × 3 mm, yielding an excitation power density of 9.33 W cm^−2^. To estimate the excitation power dependence of UCL, laser power was varied as per manufacturer's specifications. UCL was detected with a 1.5 nm detector bandwidth, a step size of 2 nm, and a dwell time of 1 ms.

UCL LT measurements were performed at 980 nm excitation at maximum laser power, *i.e.* 2 W (33.3 W cm^−2^), operating in pulsed mode; pulse width modulation provided by a control box (PM-1, Edinburgh Instruments Ltd). UCL decay kinetics were measured at 650 nm using a 1.5 nm detection bandwidth set to 10 000 peak counts, with a dwell time of 2 ms at 2000 channels. LT decay curves were analysed using the ExpDec2 (two-term exponential fit) function in Origin 2025, using a variance-weighted fitting routine. As the UCL decay curves exhibited a multiexponential decay behaviour, arising from multiple populations of Er^3+^ ions, multi-exponential fitting was required to quantify the UCL lifetimes. Multi-exponential fitting provides a more comprehensive measure of total photon emission over time and enables to consider the decay kinetics of multiple populations of excited-state emissive Er^3+^ ions, some of which undergoing FRET, some being quenched, and some being unaffected by the environment.^33^ The resultant two-term exponential fits were used to calculate an amplitude-weighted average LT (*τ*), by integrating the normalised counts, which is reported as a single *τ* value.^34^

For FRET-based biosensors, a high FRET efficiency is essential to achieving dynamic readout *via e.g.* modulation of UCNP luminescence or emission lifetime. FRET efficiency can be studied by both LT-based measurements and by quenching (*i.e.* reduction) in UCL intensity of donor UCNPs.^35^

LT-based FRET efficiency can be expressed as the percentage reduction in UCNP LT, which reflects the energy transfer between the donor UCNP and the acceptor dye, this was calculated *via*eqn (1):1
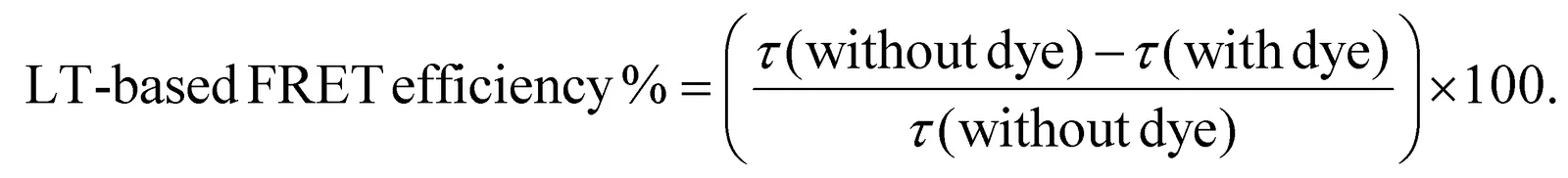


Where *τ* with subscripts denotes the LT of UCNPs (donors) in the presence or absence of AF647 dye molecules (acceptors) repectively.^36,37^

Intensity-based FRET efficiency estimates *via* reduction in total intensity were estimated as per eqn (2).2
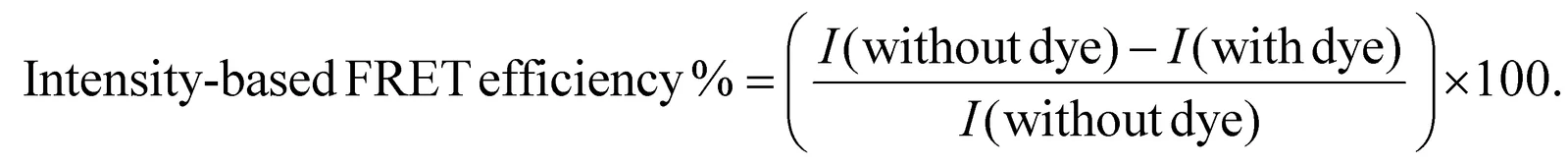


Where *I* with subscripts denotes the UCL intensity of UCNPs (donors) in the presence or absence of AF647 dye molecules (acceptors) respectively. Emission intensities were normalised to the value at 540 nm, and the overlapping red emission of AF647 was subtracted to determine changes in donor quenching. Estimates were based on both integrated intensity of the red emission band (636–700 nm) and peak intensity comparisons at specific wavelengths.

#### 2.2.6. Zeta potential and dynamic light scattering (DLS) measurements

The DLS and zeta potential of PEI-UCNPs was measured using a Malvern Zetasizer Nano Zs ZEN3600 instrument with backscatter detection at a 173° scattering angle. The refractive index of water (1.330) was used for the dispersant. All samples were sonicated for 30 min *via* sonic bath before measurement and measured in a folded capillary zeta cell (DTS1070, Malvern Panalytical Ltd).

#### 2.2.7. UV-Vis measurements

For molecular loading of AF647 onto the PEI-UCNPs, the supernatant (∼1 mL) was collected by first centrifuging the PEI-UCNPs. Subsequently, the concentration of unbound AF647 molecules was analysed using a Cary-60 UV-Vis spectrometer across a wavelength range of 200–800 nm. Measurements were performed using disposable cuvettes with a 1 cm optical path length. The concentration of unbound AF647 in the supernatant was calculated *via* a standard calibration curve (see SI Fig. S1).

#### 2.2.8. TEM measurements

Transmission electron microscopy (TEM) was performed on a Tecnai TF 20 X-TWIN (FEG) microscope, operating at 200 kV, at AGH University of Krakow. Before the measurements, UCNPs suspensions were transferred onto carbon film-coated TEM copper grids *via* drop casting method. Size measurements were conducted using ImageJ, with pixel dimensions converted to nanometres using the image scale bar as calibration. Feret diameters were used due to the irregular, non-spherical shape of UCNPs where the Maximum Feret diameter (max) is the largest distance measured between any two tangent parallel lines and the Minimum Feret diameter (min) is the smallest distance measured between any two tangent parallel lines.^38^ 50 individual UCNPs were measured from multiple TEM images obtained from a single grid for each sample. Particles were randomly selected, avoiding particles that were visibly conjoined and could not be reliably assigned.

#### 2.2.9. PXRD measurements

Dry powder samples were prepared by decanting ∼5 mg of UCNPs in H_2_O into a glass vial and heating to 120 °C (hotplate temperature) for ∼2 h until H_2_O was fully evaporated. The dry residue was scraped with a clean spatula within the vial to form a white powder. The samples were then placed into a Kapton-backed multi-well sample holder. PXRD measurements were obtained using a D8 Discover (Bruker Corporation) X-ray diffractometer using a Cu K*α* X-ray source (*λ* = 1.5406 Å) and scanning 2*θ* angles between 10° and 90° in 0.017° increments at a rate of 0.5 s per increment. After acquisition, the background of each PXRD pattern was removed using the background subtraction function of *Diffrac.eva* software (Bruker Corporation). PXRD peaks were indexed by comparison to reference pattern data. The cubic (α-phase) crystal lattice parameter, *a*, was calculated from the average crystal lattice parameter estimate of eight major peaks (*a*_*hkl*_) *via*eqn (3) and (4):3
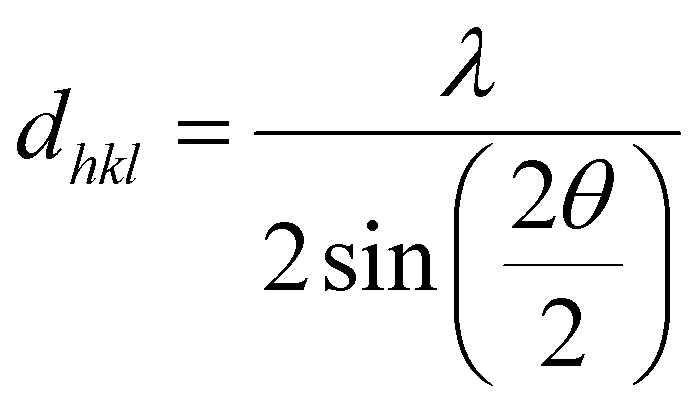
4
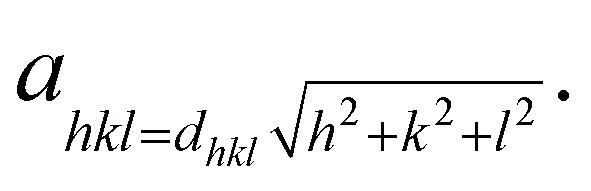


Where *d*_*hkl*_ is the interplanar spacing, *λ* is the X-ray wavelength, 2*θ* is the two-theta angle of each peak and *hkl* are the Miller indices corresponding to each peak. Uncertainties for *a* are provided by calculating *a* from a number of peaks and then calculating average and standard deviation.

## 3. Results and discussion

### 3.1. Structure and morphology

The resultant PEI-UCNPs of varying PEI MWs (1.2, 10 and 25 kDa as stated by the manufacturer) are referred to herein as 1.2 kDa UCNPs, 10 kDa UCNPs, and 25 kDa UCNPs and collectively referred to as PEI-UCNPs. Crystal structures were verified by PXRD (see Fig. 3); all the diffraction peaks of NaYF_4_:Yb,Er UCNPs correspond with test card data for cubic (α-phase) NaYF_4_. The crystal lattice parameter, α, was estimated to be 5.51 ± 0.02 Å for 25 kDa UCNPs, 5.51 Å ± 0.03 for 10 kDa UCNPs, and 5.50 ± 0.02 Å for 1.2 kDa UCNPs. Notably, 25 kDa UCNPs exhibited minor additional peaks (highlighted by * in Fig. 3), which may be indicative of the presence of a small amount of hexagonal phase NaYF_4_ in this sample, of which the origin is unclear. These crystal lattice parameters are similar to the 5.47 ± 0.02 Å previously reported by Nampi *et al.*, (2019)^39^ for 25 kDa UCNPs; indicative of good inter-study inter-laboratory reproducibility for this PEI-UCNP synthesis.

**Fig. 3:**
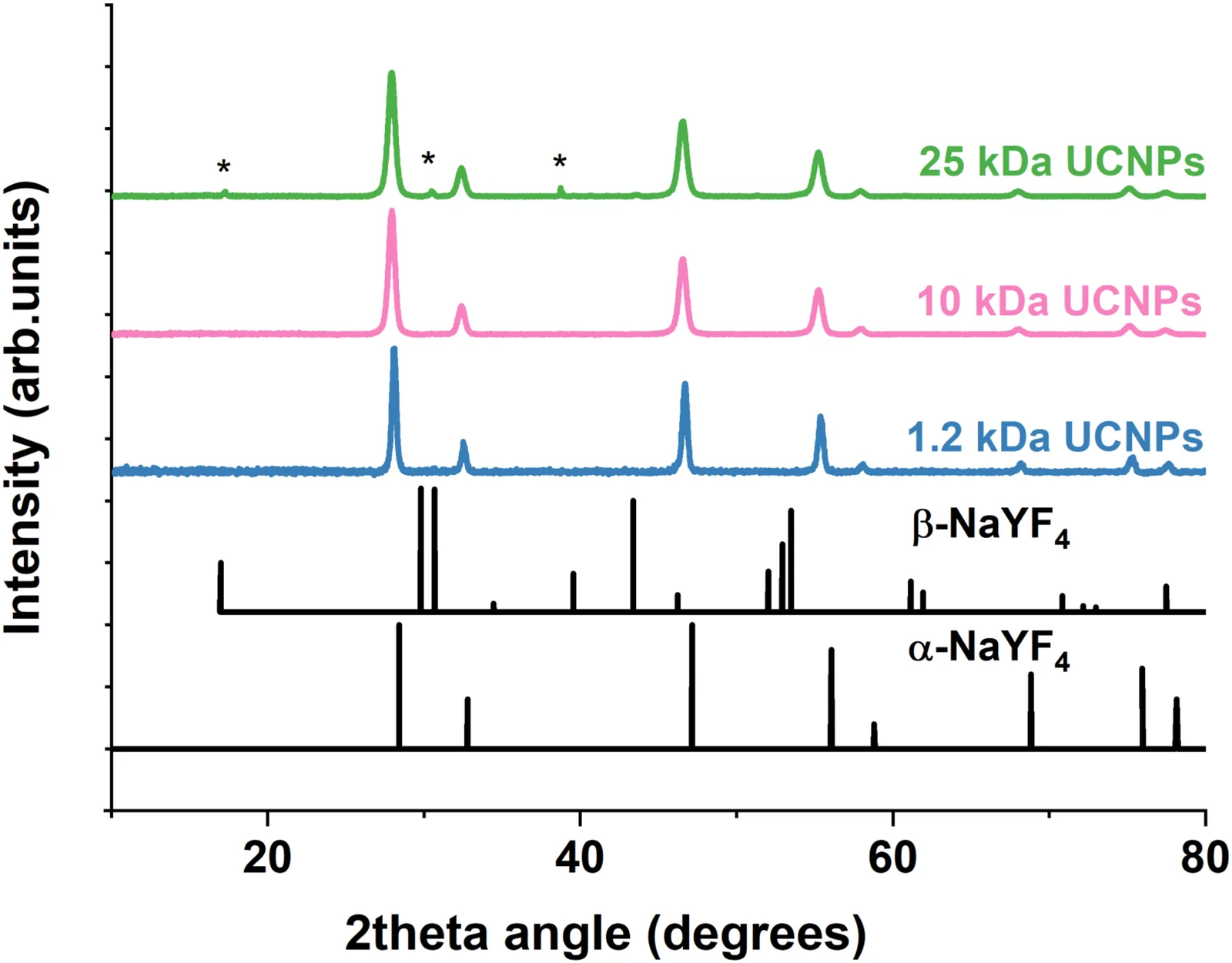
PXRD analysis of UCNPs with different PEI molecular weights. Against cubic reference card and hexagonal reference card. Peaks corresponding to the hexagonal crystal structure are highlighted by (*).

The high-resolution (HR)-TEM images of resultant PEI-UCNPs are shown in Fig. 4(a) alongside the maximum and minimum Feret diameters in Fig. 4(b) and (c). Increasing PEI molecular weight resulted in reduced average minimum Feret diameter (see Table 2). However, when considering the total area (min × max), 10 kDa UCNPs result in the smallest particles, but only ∼3.5% smaller than that of 25 kDa UCNPs. This could be attributed to steric hindrance associated with polymer MW (a proxy for polymer chain length); higher PEI MW, *i.e.* longer PEI chain lengths, are likely to exert greater steric hindrance around growing nuclei, which could lead to more controlled particle growth upon nucleation, resulting in smaller PEI-UCNPs. However, this hypothesis requires further investigation. Statistical analysis confirmed that 1.2 kDa PEI-UCNPs were significantly larger than both 10 and 25 kDa PEI-UCNPs (one-way ANOVA with Tukey's multiple-comparisons test, *p* < 0.001), while no significant difference was observed between 10 and 25 kDa UCNPs. Smaller polymer chain lengths with a lower degree of steric hindrance (1.2 kDa) may allow larger particles to grow, as shown by the larger size distributions observed.^40,41^ The average Feret diameter for 25 kDa UCNPs was 25.1 ± 7.1 nm, which is very close to the diameter of 25 kDa UCNPs produced by Nampi *et al.*, (2019)^39^*i.e.* 20 ± 4 nm. It is worth noting that Nampi *et al.* utilised a rough estimation approach *via* a circle fitting algorithm, and so likely their measurements do not reflect true nanoparticle diameter.^39^ These very similar results serve to highlight excellent inter-study inter-lab repeatability of the PEI UCNP synthesis.

**Fig. 4:**
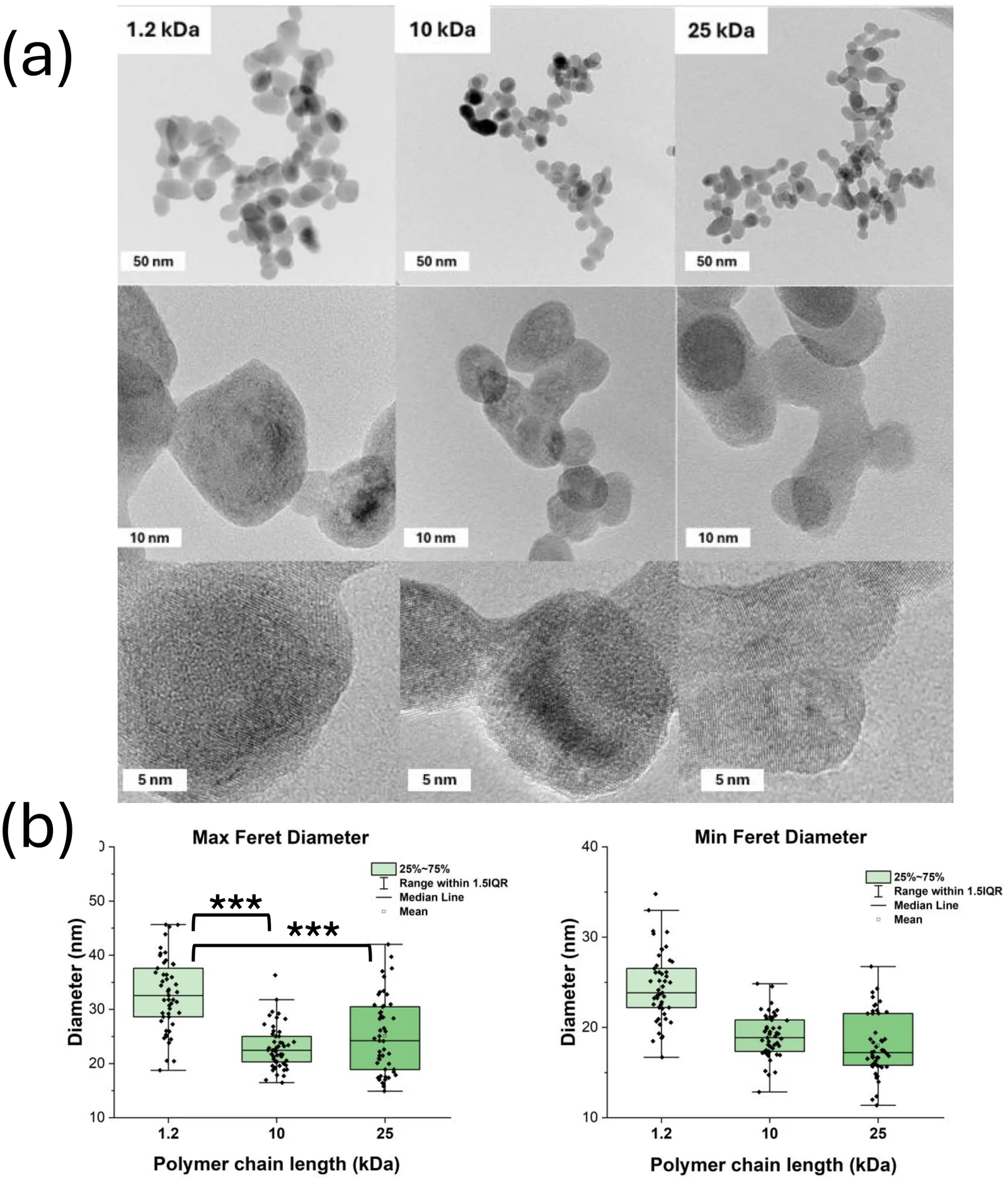
Analysis of PEI-UCNP diameters. (a) HR-TEM images of NaYF_4_:Yb,Er PEI-UCNPs. Top row: scale bar is 50 nm. Mid row: higher magnification – scale bar is 10 nm. Bottom row: highest magnification −5 nm scale bar. (b) Maximum and minimum Feret diameters (*n* = 50). Statistical significance denoted by *.

**Table 2:** Estimated Feret diameters and corresponding areas for PEI-UCNPs

PEI molecular weight (kDa)	Max. Feret diameter (mean ± SD) (nm)	Min. Feret diameter (mean ± SD) (nm)	Total area (min. × max.) (nm^2^)
**1.2**	33.0 ± 6.4	24.2 ± 3.8	806.4 ± 218.1
**10**	23.0 ± 4.1	19.0 ± 2.4	439.6 ± 100.3
**25**	25.1 ± 7.1	18.0 ± 3.4	460.0 ± 177.1

A notable feature is that some PEI-UCNPs appear conjoined (see SI Fig. S2). For the 1.2 kDa UCNPs, several conjoined particles were observed. Inspection of the crystal lattice *via* HR-TEM (see SI Fig. S2) indicates that there are two growth modes: (1) individual crystals growing independently, before conjoining with mismatched lattices (see SI Fig. S2(a) and (b)) and (2) a single crystal lattice resembling an elongated single-crystal morphology – see SI Fig. S2(c) and (d) – which indicates capital growth in a single direction.^42,43^ This could be attributed to the steric hindrance of the longer polymer chain on the particle surfaces.

DLS measurements revealed that PEI molecular weight also strongly influences the hydrodynamic behaviour of the UCNPs in aqueous media. All samples exhibited similar positive zeta potentials (∼22–24 mV), indicating moderate colloidal stability and that PEI is present on the surface of the PEI-UCNPs. However, substantial differences in hydrodynamic diameter were observed: 1.2 kDa-UCNPs displayed the smallest average hydrodynamic diameter (127.7 ± 15.8 nm) and lowest polydispersity index (PDI = 0.15), suggesting improved dispersion and reduced aggregation in water. In contrast, 10 and 25 kDa UCNPs exhibited considerably larger hydrodynamic diameters (488.4 ± 149.2 nm and 413.0 ± 60.1 nm, respectively) alongside higher PDIs (0.34 and 0.29 respectively), consistent with increased aggregation and broader size distributions when in suspension. It is worth noting that the hydrodynamic diameters are considerably larger than the measured TEM values, further supporting particle aggregation in water, however this would also be expected as DLS measures the solvated hydrodynamic diameter rather than the dry particle sizes measured by TEM.^44^

ICP-MS results (SI Tables S2 and S3) confirmed that all PEI-UCNPs produced were of a Y^3+^ : Yb^3+^ : Er^3+^ stoichiometry consistent with the intended ratio of (0.78 : 0.20 : 0.02) (generally reported as optimal for Yb^3+^ : Er^3+^ upconversion emission).^45–47^ This confirmed that PEI MW did not significantly alter the incorporation of photo-active precursors into the PEI-UCNPs.

### 3.2. UCL and LT analysis

Steady-state UCL spectra, see Fig. 5(a), exhibited an intense red emission band (∼640–690 nm, ^4^F_9/2_ → ^4^I_15/2_ transition), with a weaker green emission band at 540 to 560 nm (^2^H_11/2_, ^4^S_3/2_ → ^4^I_15/2_ transition).^39^ The high red-to-green emission ratio is a distinct characteristic of cubic-phase PEI-UCNPs and is observed in some prior studies.^19,39,48,49^ We posit that this red emission bias may be due to the NH vibrations (3100–3500 cm^−1^)^50^ introduced by the many amine groups of PEI; NH vibrations have a similar vibrational energy to OH vibrations in water (3300–3700 cm^−1^), which are known to quench the higher-energy green UCL emission of the ^2^H_11/2_, ^4^S_3/2_ → ^4^I_15/2_ transition, increasing the relative contribution of red emission.^15,51,52^ This is supported by comparison with water-dispersible PVP (polyvinylpyrrolidone)-UCNPs reported by Birch *et al.* 2023,^14^ which have an identical elemental composition, but contains no NH groups within the PVP coating. The calculated red-to-green emission ratio of the PVP-UCNPs was 1.15, compared to 20.1–22.9 for the PEI-UCNPs investigated here, This substantial difference is consistent with a possible contribution from PEI-associated NH vibrations causing a red emission bias. However, this mechanism is proposed rather than directly demonstrated here, and other differences in the PEI surface environment may also influence green and red emission pathways. To further investigate the influence of UCL quenching, we conducted experiments suspending the PEI-UCNPs in heavy water (D_2_O).^15,53–55^ For all PEI-UCNPs, the green UCL was strongly enhanced by 248–365% in D_2_O, whereas the red UCL was enhanced by only 38–64% in D_2_O (see Fig. 6). This indicates that green emission is quenched to a proportionally greater degree than red emission. However, the red-green UCL ratio was still much larger than that commonly reported for cubic-phase NaYF_4_:Yb,Er UCNPs with non-PEI surface ligands.^10,56,57^ 25 kDa UCNPs and 10 kDa UCNPs showed a greater UCL enhancement in D_2_O than 1.2 kDa UCNPs, likely due to increased steric hindrance from the higher-MW PEI, which may provide greater shielding, which would in turn, reduce O–H-mediated surface quenching. The 1.2 kDa UCNPs were still several times brighter overall, suggesting their higher UCL intensity is not solely determined by water-mediated surface quenching. The aggregation observed by DLS may also contribute to the differences in UCL intensity between PEI molecular weights through light scattering and excitation efficiency.^58^ However, aggregation does not directly follow the UCL trend observed, with 10 kDa UCNPs exhibiting the greatest hydrodynamic diameter, whereas 25 kDa UCNPs show the lowest UCL intensity. Therefore, aggregation may contribute to, but cannot account for the observed differences in UCL. For all PEI UCNPs, UCL LT also increased in D_2_O (see Fig. 6, SI Tables S4 and S5). Overall, these results demonstrate that whilst aqueous quenching does affect these PEI-UCNPs, there may also be another factor resulting in the unusual high red/green ratio of PEI UCNPs, *e.g.* NH vibrations from PEI (see section 4 for further discussion).

**Fig. 5:**
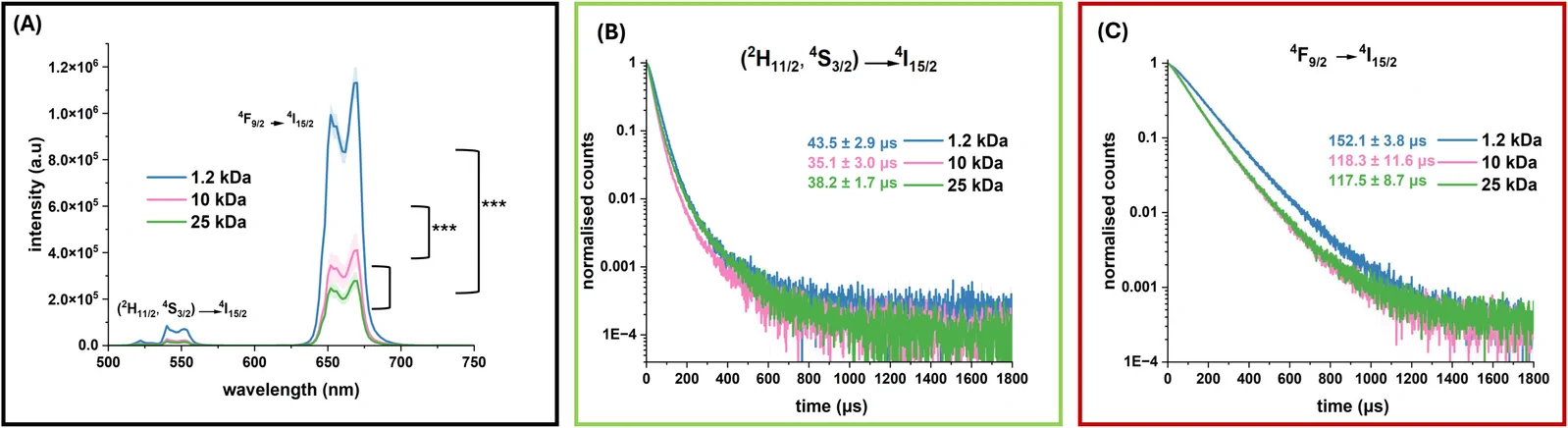
Lower MW PEI enhances UCL and LT. (A). Steady-state UCL spectra of PEI-UCNPs in DI H_2_O (10 mg mL^−1^, 980 nm excitation). 1.2 kDa UCNPs exhibited ∼4× greater UCL intensity than 25 kDa UCNPs (integrated area). Shaded regions represent standard error of 3 independently produced samples. Statistical significance was assessed using ordinary one-way ANOVA followed by Tukey's multiple-comparisons test. *** denotes *p*-values <0.001. Comparisons without an asterisk were not statistically significant (*p* ≥ 0.05) UCL lifetime measurements acquired under 980 nm excitation recorded at (B) 540 nm and (C) 650 nm (Edinburgh Instruments FS5-V2).10 kDa and 25 kDa UCNPs have similar LTs well within margin-of-error, whereas the 1.2 kDa UCNPs exhibit a 29% greater LT. All measurements were conducted at a particle concentration of 10 mg mL^−1^ in DI water. The reported *τ* values represent the mean ± standard deviation of 3 independently prepared samples. LT fits presented in SI section S8 and statistical analysis is shown in SI section S9.

**Fig. 6:**
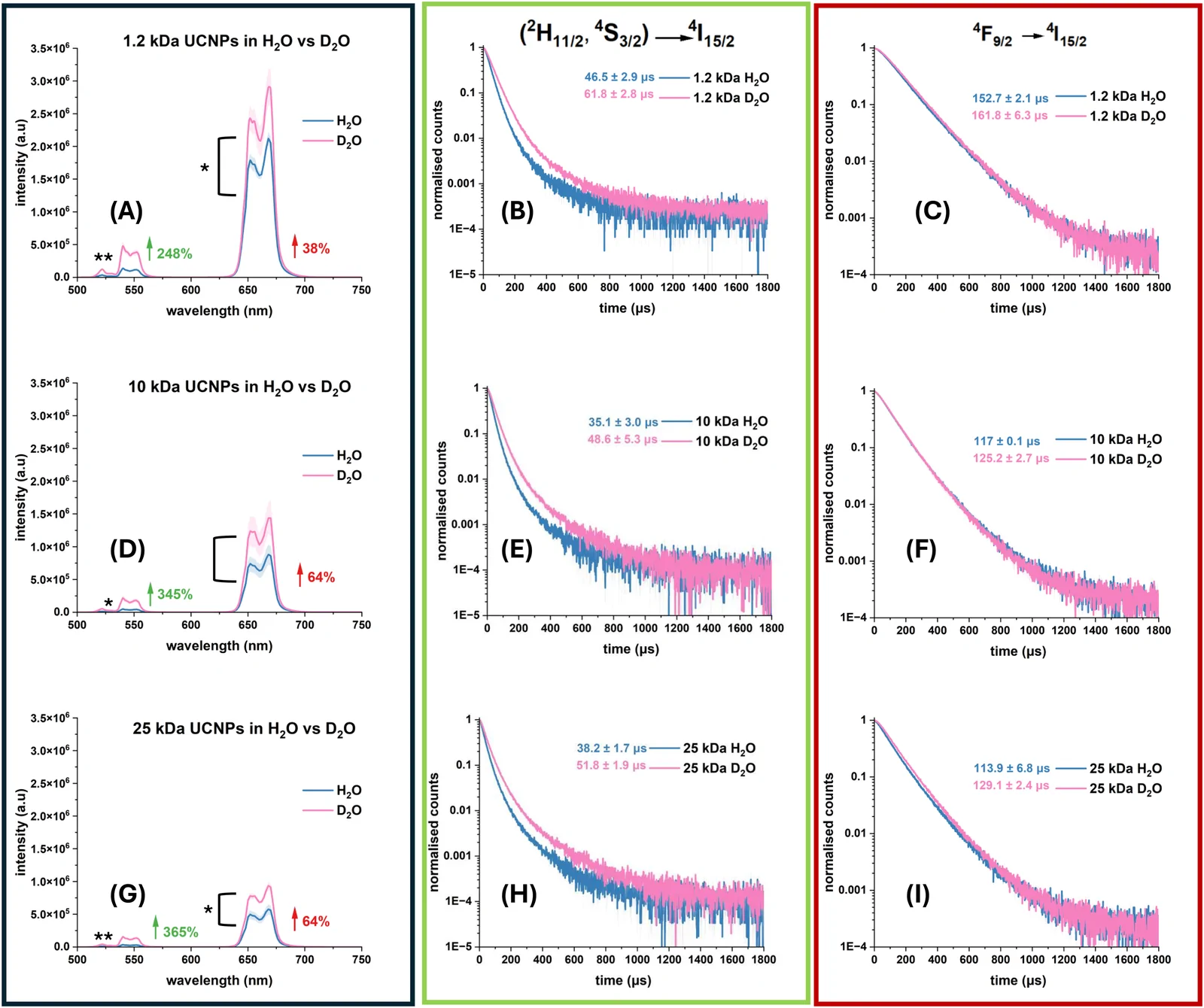
PEI MW influences vibrational quenching of UCL by H_2_O and D_2_O. (A, D and G) Steady-state UCL spectra of 1.2, 10 and 25 kDa PEI-UCNPs in H_2_O and D_2_O (10 mg mL^−1^). D_2_O suspension enhanced emission across all samples with a proportionally greater increase in green emission than red emission. 980 nm excitation. The shaded area is standard error of three independently produced samples. Statistical significance between H_2_O and D_2_O was assessed at 540 nm (green emission) and 670 nm (red emission) using ordinary one-way ANOVA followed by Tukey's multiple-comparisons test. * denotes *p* < 0.05; ** *p* < 0.01. Comparisons without an asterisk were not statistically significant (*p* ≥ 0.05). Corresponding UCL LT measurements collected at (B, E and H) 540 nm, and (C, F and I) 650 nm showing increased LTs in D_2_O due to reduced UCL quenching. LT fits presented in SI section S8 and statistical analysis is shown in SI section S9.

The UCL population pathways were further investigated by measuring the excitation power dependence of UCL. In upconversion processes, the UCL intensity (*I*) correlates with *I* = *P*^*n*^, where *P* is the excitation power and *n* is the number of photons required for upconversion, respectively.^10^ From this, the number of photons involved in a non-saturated UCL process can be derived from the gradient of a log–log plot of excitation power *vs*. UCNP emission (see Fig. 7).

**Fig. 7:**
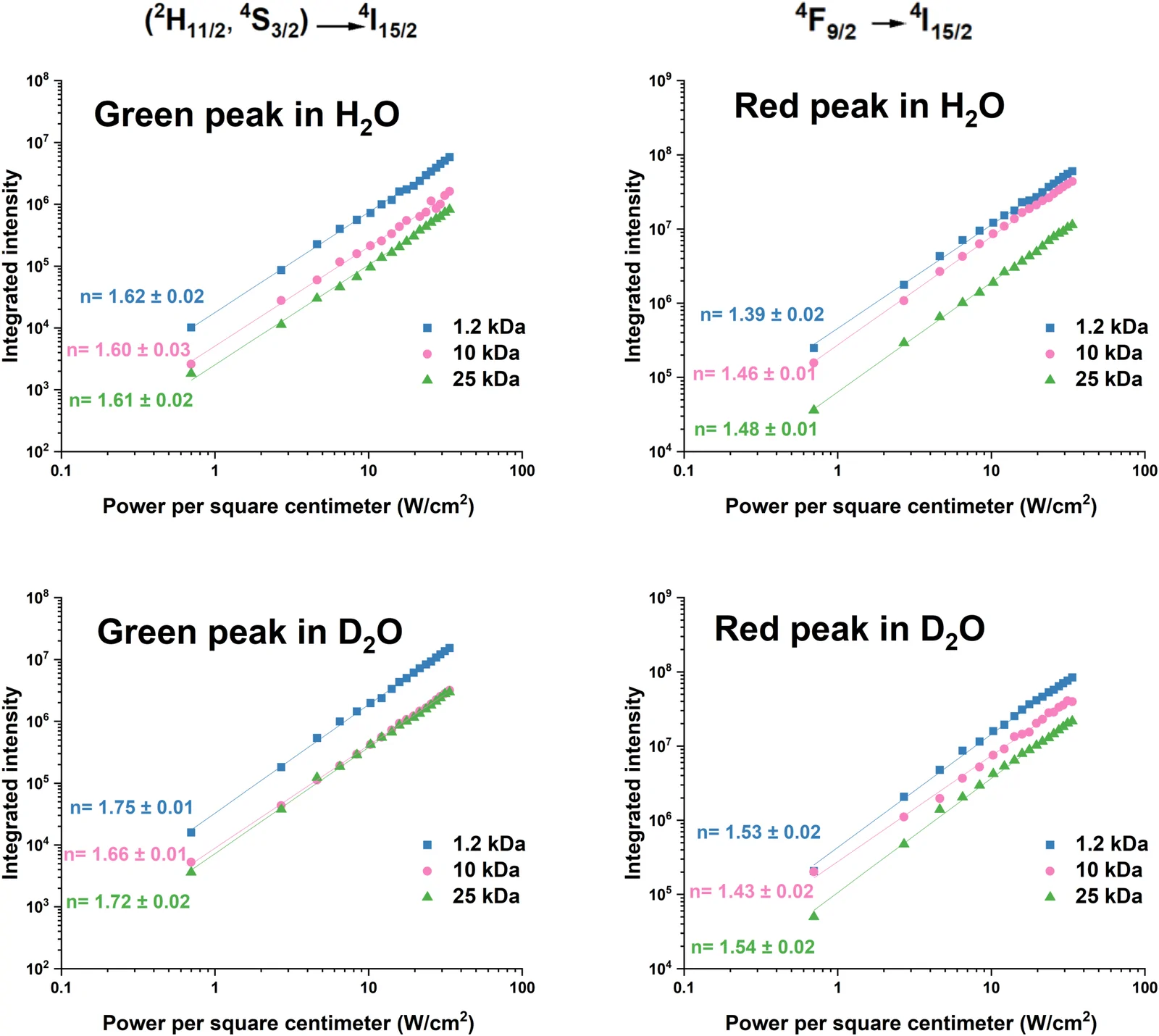
Excitation power dependence *vs*. steady-state state UCL intensity for PEI-UCNPs. 980 nm excitation, 10 mg mL^−1^ in H_2_O and D_2_O. The green peak corresponds to integrated intensity between 516–566 nm, and the red peak between 626–706 nm. Errors are derived from uncertainties straight line fits.

In H_2_O, *n* for the ^2^H_11/2_, ^4^S_3/2_ → ^4^I_15/2_ transition (green, 516 to 556 nm) remained relatively consistent for all PEI-UCNPs, with *n* values ranging from 1.60 ± 0.02 to 1.62 ± 0.02, indicating similar green upconversion processes for all PEI-UCNPs in H_2_O. *n* increased in D_2_O, ranging from 1.66 ± 0.01 (10 kDa PEI UCNPs) to 1.75 ± 0.01 (1.2 kDa UCNPS), indicating improved green UCL in D_2_O due to reduced UCL quenching *via* OH vibrations. This effect was most pronounced for 1.2 kDa UCNPs (1.62 ± 0.02 to 1.75 ± 0.01) and 25 kDa UCNPs (1.61 ± 0.02 to 1.72 ± 0.02). In contrast, for the ^4^F_9/2_ → ^4^I_15/2_ transition (red, 626–706 nm), *n* values were generally lower (corresponding to lower energy upconversion) and showed a weaker dependence on solvent environment. In H_2_O, *n* values ranged from 1.39 ± 0.02 (1.2 kDa UCNPs) to 1.48 ± 0.01 (25 kDa UCNPs). In D_2_O, *n* increased to 1.53 ± 0.02 (1.2 kDa UCNPs) and 1.54 ± 0.02 (25 kDa UCNPs). The exception was 10 kDa UCNPs, which exhibited a slight decrease from 1.46 ± 0.02 in H_2_O to 1.43 ± 0.02 in D_2_O. The differing behaviours of the green and red emission pathways suggest that PEI MW influences surface quenching by OH groups, while the PEI itself, including NH vibrations, may also affect the upconversion process.

### 3.3. Adsorption of a model fluorescent dye AF647 to PEI-UCNPs

A model fluorescent dye, AF647 (see Fig. 2c) was utilised to explore how PEI-UCNPs can serve as FRET donors. AF647 was selected for several reasons. (1) The absorption of AF647 overlaps with the red Er^3+^ emission (see Fig. 2d). (2) AF647 is water-soluble, with good reported photostability, and its optical properties are relatively insensitive to pH. (3) AF647 has a reported moderate fluorescence quantum yield of 33% (in PBS 50 mM potassium phosphate, 150 mM NaCl, pH 7.2 at 22 °C).^59,60^ (4) AF647 is available as an NHS conjugate, which provides a route to binding AF647 to PEI-UCNPs (electrostatically or covalently) *via* the amine groups of PEI.

The adsorption of AF647 to the PEI-UCNPs made from the various PEI MW's was studied in DI water, with the AF647-modified UCNPs forming a blue-tinted solution. The loading efficiency for all PEI-UCNP formulations referred to as PEI-UCNP@AF647 ranged between 60–46%, indicating successful attachment of AF647 to PEI-UCNPs (see SI Table S1). To further investigate the differences in AF647 loading between PEI molecular weights, the primary amine content of the PEI-UCNPs was investigated (SI section S3). Primary amine content was 0.280, 0.439 and 0.392 mM mg^−1^ for 1.2, 10 and 25 kDa PEI-UCNPs, respectively. Interestingly, primary amine content did not directly correlate with AF647 loading efficiency; despite having a greater primary amine content than the 1.2 kDa UCNPs, the 25 kDa UCNPs exhibited the lowest AF647 loading. This suggests that AF647 loading is not determined solely by the total number of primary amines detected, but may also depend on the accessibility of these groups to AF647 and electrostatic interactions within the PEI surface layer. Differences in polymer chain conformation and steric hindrance with increasing PEI molecular weight may therefore contribute to the observed differences in dye loading.

### 3.4. PEI MW *vs*. observed FRET efficiency in H_2_O

AF647 alone showed no emission under 980 nm excitation (null data – not shown). From Fig. 8(a, c, e and g), it can be seen that PEI-UCNPs@AF647 exhibited characteristic AF647 fluorescence (peak ∼680–750 nm) when excited at 980 nm, confirming sensitisation by FRET from the UCNPs. PEI-UCNPs@AF647 loaded with a higher initial concentration of AF647 exhibited decreased UCL at wavelengths overlapping with AF647 absorption, and increased AF647 emission (see Fig. 8a, c, e, and 2d). UCL lifetimes were reduced when AF647 was loaded, with this effect increasing at higher initial AF647 concentration (see Fig. 8b, d and f). This further confirmed FRET between UCNPs (donor) and AF647 (acceptor). The greatest reduction in lifetime was exhibited by 25 kDa PEI-UCNPs@AF647 with an initial loading concentration of 10 µM AF647, corresponding to a LT-based FRET efficiency of 11% (see SI Table S6).

**Fig. 8:**
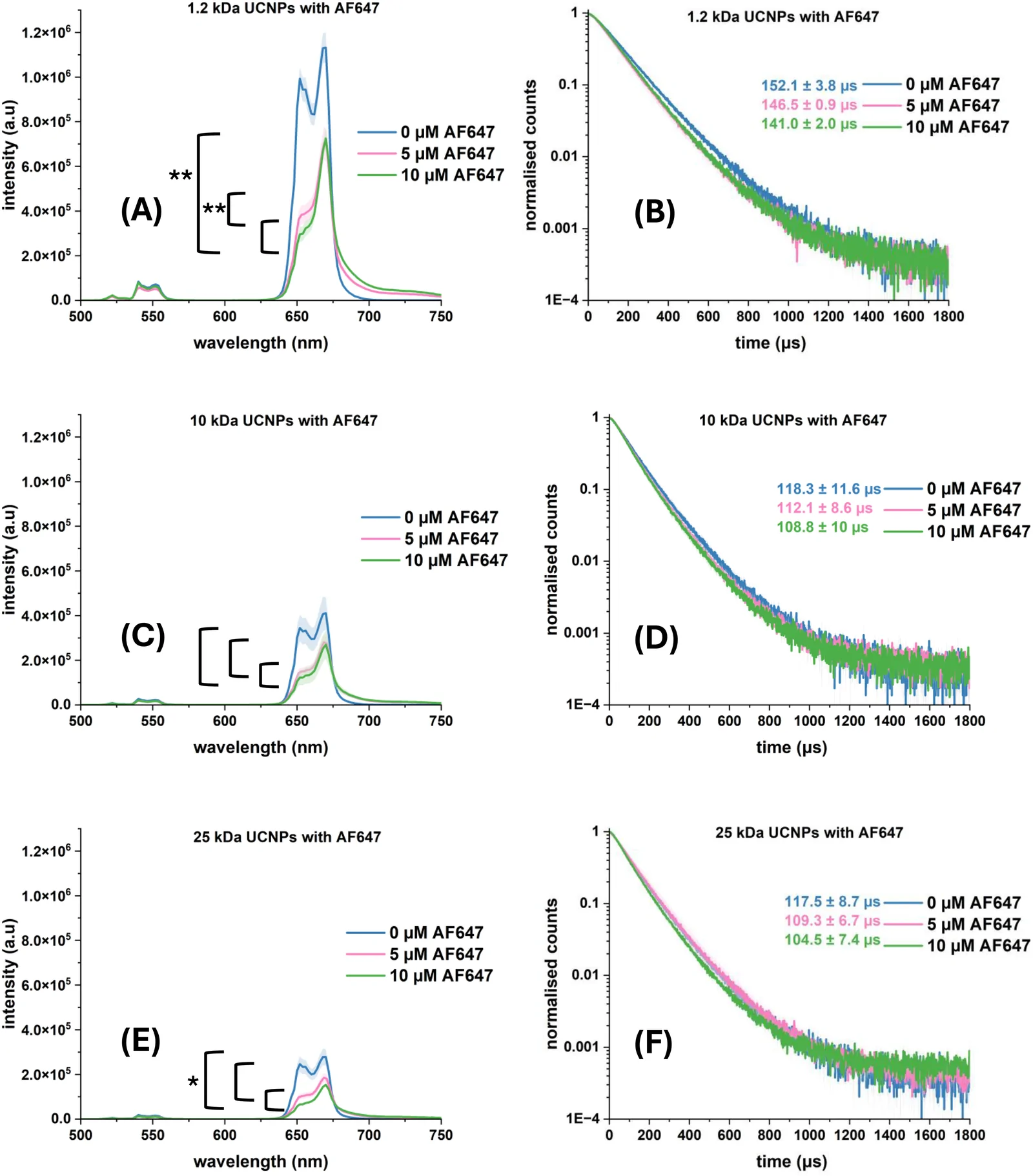
PEI-UCNPs act as FRET donors for acceptor fluorophore AF647 in DI H_2_O. (A, C and E) Steady-state UCL spectra demonstrating FRET between PEI-UCNPs (donor) and AF647 (acceptor) at initial AF647 loading concentrations of 5 μM and 10 μM. The increased emission at ∼680–750 nm can be attributed to AF647 excitation *via* UCNPs. Measurements in DI water, 980 nm excitation, 10 mg mL^−1^ UCNPs. Shaded regions represent standard error from repeated measurements of 3 independently synthesised samples. Statistical significance was assessed within each PEI molecular weight using ordinary one-way ANOVA followed by Tukey's multiple-comparisons test. For the 1.2 kDa PEI-UCNPs, Tukey-adjusted *p*-values were 0.009 for UCNPs alone *versus* 5 μM AF647, 0.009 for UCNPs alone *versus* 10 μM AF647, and 0.99 for 5 *versus* 10 μM AF647. For the 10 kDa PEI-UCNPs, the corresponding *p*-values were 0.36, 0.27 and 0.97, respectively. For the 25 kDa PEI-UCNPs, the corresponding *p*-values were 0.096, 0.027 and 0.59, respectively. (B, D and F) Corresponding UCL LT measurements collected at 980 nm excitation and measured at 650 nm for varying PEI molecular weights. *τ* values are reported as mean ± standard deviation of measurements from three of independently synthesised samples. LT fits presented in SI section S8 and statistical analysis shown in SI section S9.

The greater FRET response observed for 25 kDa PEI-UCNPs does not appear to arise simply from increased dye loading or primary amine content. Primary amine content was 0.280, 0.439 and 0.392 mM mg^−1^ for 1.2, 10 and 25 kDa PEI-UCNPs, respectively, while 25 kDa UCNPs exhibited the lowest AF647 loading efficiency. Therefore, neither total primary amine content nor AF647 loading directly correlated with the observed FRET response. Instead, differences in the PEI surface environment and spatial distribution of AF647 may influence donor–acceptor proximity and consequently FRET efficiency. The smaller particle dimensions of the higher-MW PEI-UCNPs may also increase the proportion of Er^3+^ donors sufficiently close to the particle surface to undergo FRET.^35^ Differences in aggregation may additionally influence FRET by altering UCNP-AF647 proximity and dye accessibility, and therefore represents another potential contribution to the MW-dependent FRET response.

According to Madirov and Hildebrandt,^35^ the reduction of both LT and steady-state UCL intensity should be considered when evaluating FRET efficiencies.^35^ The respective data are summarised in Table 2 and SI Table S7. LT estimates indicate that both increased PEI MW and increased AF647 dye loading increased FRET efficiency. Trends for intensity-based FRET efficiency were similar for an initial loading concentration of 5 μM AF647; however, at 10 μM, 25 kDa PEI-UCNPs@AF647 exhibit a smaller reduction in UCL, despite having the greatest LT reduction (see peak-intensity comparisons in SI Tables S8 and S9). This is indicative of multiple pathways influencing PEI-UCNP emission and further highlights the importance of reporting both LT-based and intensity-based FRET efficiencies. The 25 kDa UCNPs exhibited 78% intensity-based FRET efficiency – this is similar to the ∼71% intensity-based FRET efficiency reported by Wang *et al.*, 2025 for similar PEI-UCNPs conjugated to chlorin e6 photosensitizer.^48^

### 3.5. PEI molecular weight *vs*. observed FRET efficiency in D_2_O

To further investigate the effect of PEI MW and quenching dynamics on FRET efficiency of PEI-UCNPs, the UCL and LT were recorded in D_2_O (see Fig. 9). LT-based FRET efficiency was greater in D_2_O than in H_2_O (see Table 3) with 25 kDa PEI-UCNPs achieving the greatest LT-based FRET efficiency of 11% in H_2_O and 16% in D_2_O with an initial AF647 loading concentration of 10 µM (Table 3). In contrast, intensity-based FRET efficiencies showed greater variation across PEI MW and AF647 concentration, and the UCL intensity change was ∼20% higher for PEI-UCNPs in D_2_O *vs*. UCNPs in H_2_O (Table 3). The highest intensity reduction (85%) was exhibited by 1.2 kDa UCNPs for an initial 5 µM concentration of AF647 in D_2_O (see SI sections S6 and S7 for full tabulated datasets).

**Fig. 9:**
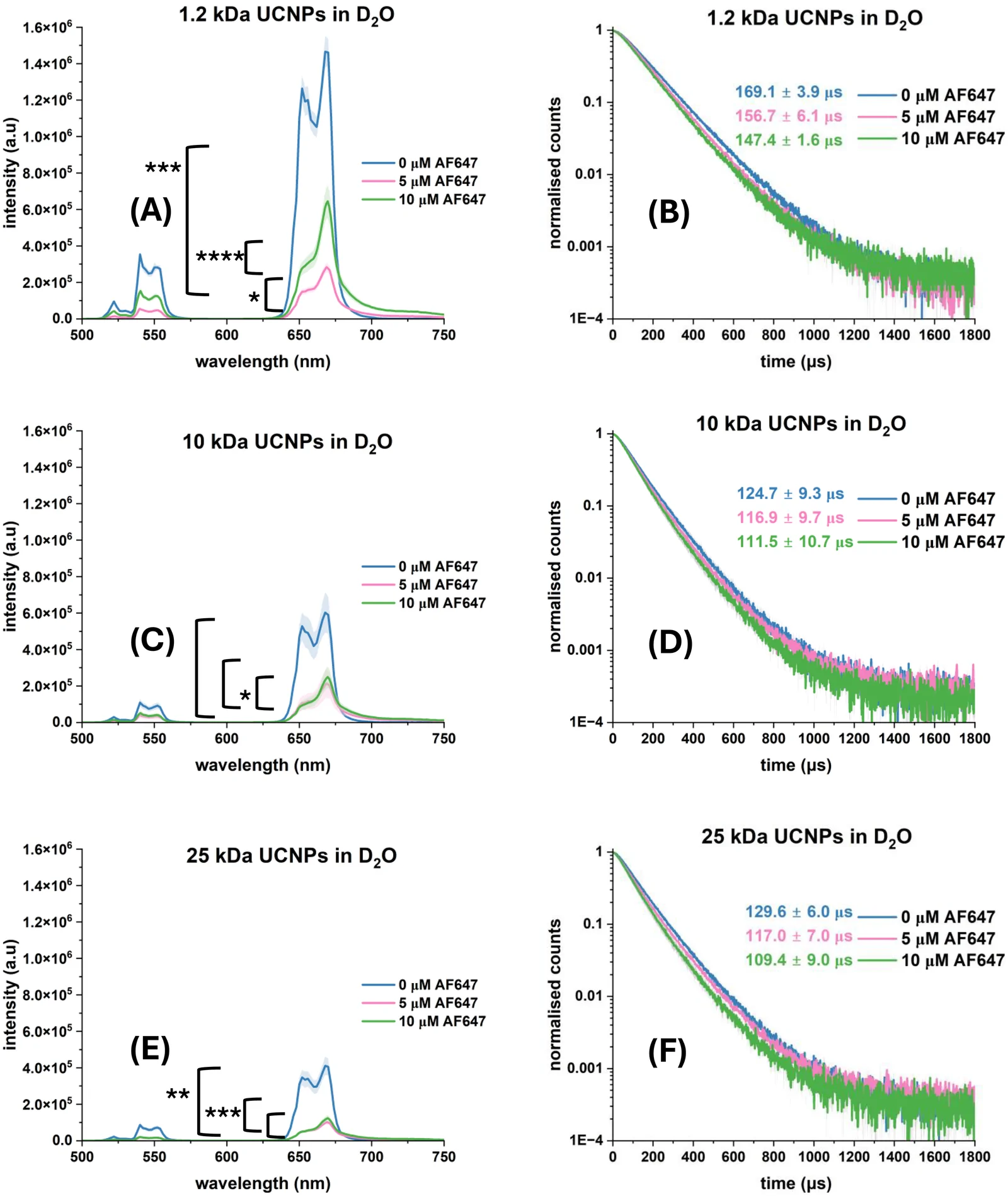
Effect of initial AF647 concentration on FRET exhibited by PEI-UCNPs in D_2_O. (A, C and E) Steady-state emission spectra demonstrating FRET between PEI-UCNPs (donor) and AF647 (acceptor) at 5 μM and 10 μM initial AF647 concentrations in D_2_O water. 980 nm excitation, 10 mg mL^−1^ UCNPs. Shaded regions represent standard error of measurements from three independently synthesised samples. Statistical significance was assessed within each PEI molecular weight using ordinary one-way ANOVA followed by Tukey's multiple-comparisons test. For the 1.2 kDa PEI-UCNPs, Tukey-adjusted *p*-values were <0.0001 for UCNPs alone *versus* 5 μM AF647, 0.0009 for UCNPs alone *versus* 10 μM AF647, and 0.032 for 5 *versus* 10 μM AF647. For the 10 kDa PEI-UCNPs, the corresponding *p*-values were 0.044, 0.061 and 0.96, respectively. For the 25 kDa PEI-UCNPs, the corresponding *p*-values were 0.0009, 0.0013 and 0.89, respectively. * *p* < 0.05, ** *p* < 0.01, *** *p* < 0.001. Comparisons without an asterisk were not statistically significant (*p* ≥ 0.05). (B, D and F) Corresponding UCL LT measurements collected at 980 nm excitation and measured at 650 nm for varying PEI molecular weights. *τ* values are reported as mean ± standard deviation of measurements from 3 independently synthesised samples. LT fits presented in SI section S8 and statistical analysis in SI section S9.

**Table 3:** Summary of Intensity and LT-based FRET Efficiencies for PEI-UCNPs@AF647 in H_2_O and D_2_O

PEI MW (kDa)	Solvent	AF647 concentration (µM)	FRET efficiency, intensity (%)	FRET efficiency, lifetime (%)
**1.2**	H_2_O	5	**57**	**3**
**10**	H_2_O	5	**57**	**5**
**25**	H_2_O	5	**88**	**7**
**1.2**	H_2_O	10	**64**	**7**
**10**	H_2_O	10	**62**	**8**
**25**	H_2_O	10	**56**	**11**
**1.2**	D_2_O	5	**85**	**7**
**10**	D_2_O	5	**73**	**6**
**25**	D_2_O	5	**82**	**9**
**1.2**	D_2_O	10	**76**	**13**
**10**	D_2_O	10	**72**	**11**
**25**	D_2_O	10	**80**	**16**

The contrast in FRET efficiency % derived from LT-based and intensity-based methods highlights the complex quenching observed in UCNP systems. While LT reduction usually directly reflects energy transfer from donor to acceptor, intensity measurements are influenced by additional processes including reabsorption, dye emission overlap and non-radiative quenching.^15,35,61,62^ On the other hand, UCNPs are a specific case of FRET donors because the FRETing energy levels are quenched and sensitised simultaneously (due to the long excitation of Er^3+^*via* Yb^3+^). Therefore, LT measurements are not ideal for quantifying FRET in UCNPs either; consequently, analysing both intensities and LT is recommended to evaluate FRET with UCNP donors.^35^ As summarised in Table 2, intensity-based efficiencies were considerable greater than LT-based efficiencies across all conditions, while the trends with PEI MW and AF647 concentration differed between the two methods. This further supports the consideration of both intensity and LT measurements in UCNP-based FRET systems. FRET efficiencies were generally greater in D_2_O compared to H_2_O, particularly for LT measurements at 10 μM AF647, indicating surface vibrational quenching by OH vibrations competing with inter-ion upconversion energy transfer in aqueous environments. This suggests that solvent-dependent quenching, alongside polymer MW and AF647 concentration, play an important role in the photophysical and FRET properties of PEI-UCNPs@AF647.^15^

The Förster radius (*R*_0_) was also estimated to provide further physical context for the FRET efficiencies observed in both H_2_O and D_2_O. Using assumed donor (Er^3+^) quantum yields of 1, 5 and 10%, calculated *R*_0_ values were approximately 3.9, 5.1 and 5.7 nm, respectively, which are similar to those reported by Pini *et al.* 2023.^63^ Across both H_2_O and D_2_O, the measured intensity-based FRET efficiencies corresponded to estimated donor–acceptor distances of approximately 2.8–5.5 nm (SI section S9), providing a reasonable distance range for the FRET observed between the PEI-UCNPs and AF647. Together with the intensity and LT measurements, these results demonstrate that FRET occurs for all three PEI-UCNP formulations, while the extent of energy transfer is influenced by PEI MW, AF647 concentration and the surrounding solvent environment.

## 4. Limitations and perspectives

This study demonstrates that PEI molecular weight plays an important role in governing the structural and photophysical properties of NaYF_4_:Yb,Er PEI-UCNPs synthesised *via* a one-pot solvothermal approach. By varying PEI molecular weight, we observed differences in nanoparticle size, luminescence intensity, surface quenching behaviour, and FRET efficiency. These findings highlight polymer molecular weight as an important parameter when designing polymer-coated UCNP systems for photonic and biomedical applications.

There are, however, several areas which require further investigation. Firstly, this study focuses on water-dispersible PEI-UCNPs where PEI is introduced *in situ* as part of the one-pot solvothermal synthesis process. Whilst convenient and reliable, the synthesis does not offer the flexibility of ligand exchange associated with other UCNP synthesis approaches. Indeed, it remains to be determined how tightly bound to the NaYF_4_:Yb,Er crystal this *in situ* PEI is. Likewise, there has been no clear study of how these PEI-UCNPs initially nucleate, form, and grow. Further, we did not explore the effect of the amount of PEI used in synthesis– this has been shown to influence UCNP syntheses, such as the PVP-assisted method.^14^ Further studies investigating how polymer molecular weight, polymer concentration, and other synthesis conditions influence UCNP nucleation, growth, and optical properties would therefore be valuable.

The three molecular weights of PEI we examined were selected due to commercial availability, and we examined PEI of only one specific batch from a single manufacturer. The exact range of PEI MWs in each commercial batch is not known – although we have provided mass spectroscopy information for each batch (see SI section S8), and size exclusion chromatography will be considered for future work. Comparison of PEI from different manufacturers or even different batches may give rise to different results. This study exclusively examined branched PEI, but there are various other forms of PEI. For example, linear PEI is available (albeit at a much higher cost); we expect that linear PEI will almost certainly exhibit different behaviour if used in UCNP synthesis. We recommend characterisation of polymers used in nanoparticle synthesis wherever possible.

In this study, FRET interactions were only explored with one single model fluorophore (AF647). Other fluorophores could have been selected to fulfil a similar purpose, *e.g.* Cy5.5,^64^ and quantum dots or biomolecular fluorophores could also be explored as FRET acceptors.^26,65^ Further studies are also required to investigate how PEI molecular weight influences the colloidal stability, biocompatibility, and cellular uptake properties of PEI-UCNPs for future biomedical applications.^39^ Overall, this work highlights the substantial experimental parameter space afforded by polymer molecular weight and provides a foundation for future optimisation of polymer-coated UCNP systems.

## 5. Conclusions

This study examined how PEI MW influences the morphology, photophysical, molecular binding, and FRET response properties of PEI-UCNPs. We note that the NaYF_4_:Yb,Er PEI-UCNPs produced were highly reproducible in terms of photophysical properties with excellent repeatability between independent syntheses. Of the three PEI MWs investigated, PEI-UCNPs synthesised with 1.2 kDa PEI exhibited a 5-fold improvement in UCL in DI H_2_O compared to 25 kDa UCNPs, with a corresponding 29% increase in UCL LT. All PEI-UCNPs exhibited greater quenching in DI H_2_O compared to D_2_O, indicating OH oscillations are a factor in their UCL when dispersed in water. Whilst the PEI acts to protect UCNPs from aqueous quenching *via* OH vibrations, we posit that PEI may also introduce a source of UCL quenching *via* NH vibrations, which may be partly responsible for the distinctive high red/green UCL ratio of PEI-UCNPs. However, further experimental work beyond the scope of this manuscript is required to confirm this. All PEI-UCNPs were capable of FRET with UCNPs as donors and a model dye as acceptor, with 25 kDa PEI-UCNPs exhibiting the greatest FRET efficiency (16% LT-based FRET efficiency; 80% intensity-based FRET efficiency, both in D_2_O) Estimated donor–acceptor distances of 2.8–5.5 nm further supported the observed FRET response. These results indicate that for the PEI-UCNPs synthesized in this study, there is a trade-off between PEI-UCNP UCL intensity and efficiency of FRET response. We note that polymer MW should be carefully considered when optimising photophysical and FRET properties of UCNPs.

## Author contributions

R. M. Synthesised all UCNPs, conducted all measurements and analysis unless stated otherwise, drafted and edited the manuscript. C. H. Assisted with upconversion luminescence and lifetime measurements and analysis. Edited and approved the manuscript. C. C. Assisted with ninhydrin assay and FTIR measurements. Edited and approved the manuscript. M. G. Conducted high resolution TEM measurements. Edited and approved the manuscript. R. P. Conducted preliminary UCL measurements. Edited and approved the manuscript. N. H. Analysed and discussed the FRET results and edited and approved the manuscript. U. R. G. Experimental resources for upconversion luminescence and lifetime measurements. Edited and approved the manuscript. L. M. Funding acquisition, conceptualisation, supervision, experimental resources, project administration. Edited and approved the manuscript.

## Conflicts of interest

There are no conflicts of interest to declare.

## Supplementary Material

NR-OLF-D6NR02308H-s001

## Data Availability

The datasets generated and/or analysed during the current study have been deposited in the University of Strathclyde's Pure research data repository at https://doi.org/10.15129/2852d360-7e2b-4504-aece-993722b2ec1f. Supplementary information (SI) is available. The Supplementary Information contains additional nanoparticle characterisation data (STEM, FT-IR, ICP-MS and MALDI-MS), ninhydrin assay results, AF647 calibration and loading studies, supplementary upconversion emission and luminescence lifetime data, statistical analyses, and Förster resonance energy transfer calculations, including spectral overlap integrals. See DOI: https://doi.org/10.1039/d6nr02308h.
