# Supplementary material for: Polyethyleneimine (PEI) molecular weight alters the photophysical and FRET properties of water dispersible NaYF4:Yb,Er upconversion nanoparticles
Source: Nanoscale. 2026 Nov 4. Online ahead of print. doi: 10.1039/d6nr02308h (PMC13752746; doi:10.1039/d6nr02308h)
Supplement: Supplementary file 1 [file NR-OLF-D6NR02308H-s001.pdf]

This journal is © The Royal Society of Chemistry

Supplementary information

Polyethyleneimine (PEI) molecular weight alters the photophysical and FRET properties of water-dispersible $NaYF_4$:Yb,Er upconversion nanoparticles.

Contents

Supplementary information 1
SI Section 1 2
Molecular loading of AF647 2
SI Section 2 3
AF647 Loading efficiency 3
SI Section 5 7
UCL and LT analysis of PEI-UCNPs 7
SI Section 6 8
SI Section 7 10
SI Section 8 12
LT data fits for UCNPs in H_2O and D_2O. 12
PEI UCNPs@5 µM AF647 in H_2O and D_2O: 13
PEI UCNPs@10 µM AF647 in H_2O and D_2O: 14
SI Section 9 15
Statistical analysis 15
SI Section 10 20
SI Section 11 24
MALDI-MS data for PEI polymers used in synthesis 24
3.5.1 1.2 kDa PEI 24
3.5.2 10 kDa PEI 25
3.5.3 25 kDa PEI 26

SI Section 1

Molecular loading of AF647

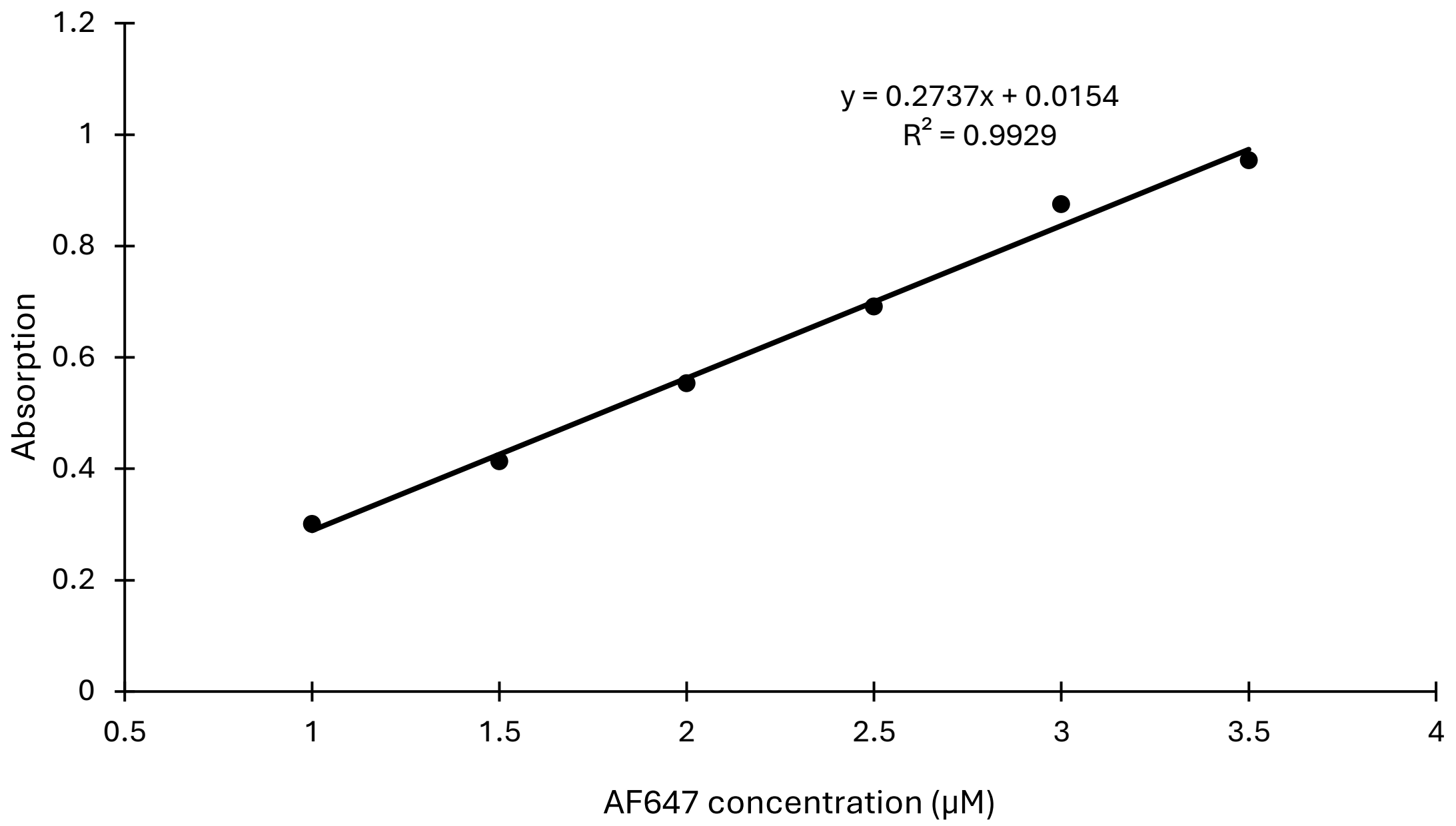

SI Figure 1. AF647 concentration vs absorption calibration using UV-Vis spectroscopy.

SI Section 2

AF647 Loading efficiency

SI Table 1. Effect of AF647 loading on the zeta potential of PEI-UCNPs and estimated loading efficiency achieved in deionised water.

**1.2 kDa UCNPs**	**AF647 concentration (μM)**	**Zeta potential (mV)**	**Loading efficiency (%)**
	**0**	+ 23.9 ± 0.6	**N/A**
	**5**	+ 23.5 ± 0.5	**60.8 ± 3.2**
	**10**	+ 23.4 ± 0.7	**54.1 ± 5.1**
**10 kDa UCNPs**	**AF647 concentration (μM)**	**Zeta potential (mV)**	**Loading efficiency (%)**
	**0**	+ 21.9 ± 0.5	**N/A**
	**5**	+ 24.6 ± 0.3	**60.6 ± 7.6**
	**10**	+32.6 ± 0.9	**54.7 ± 1.8**
**25 kDa UCNPs**	**AF647 concentration (μM)**	**Zeta (mV)**	**Loading efficiency (%)**
	**0**	+ 22.7 ± 0.3	**N/A**
	**5**	+ 24.0 ± 0.5	**52.2 ± 2.5**
	**10**	+ 23 ± 0.2	**46.2 ± 3.1**

SI Section 3

STEM image analysis

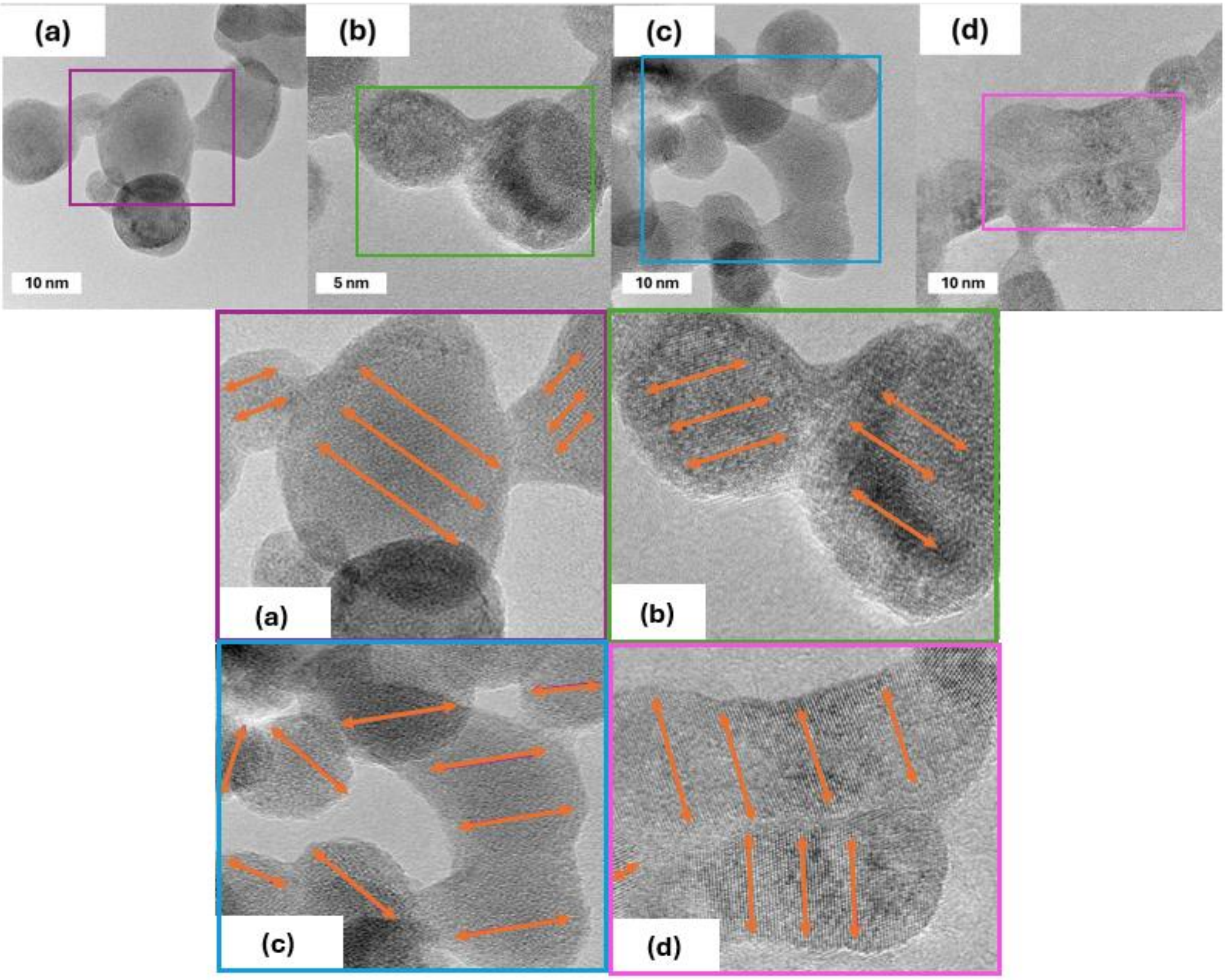

SI Figure 2. **HR-TEM images of PEI-UCNPs.** (a) 1.2 kDa UCNPs individual particles with different lattice orientations conjoined. (b)10 kDa UCNPs conjoining with differently orientated lattices and occasional growth of (c) 10 kDa UCNPs, non-descriptive shape particles with single crystal lattice. (d) 25 kDa UCNPs grow primarily into elongated, non-uniform shaped particles. Scale bar representing (a) 10 nm, (b) 5 nm, (c) 10 nm, and (d) 10 nm. Boxes are zoomed in region, but magnification is the same.

Further polymer characterisation

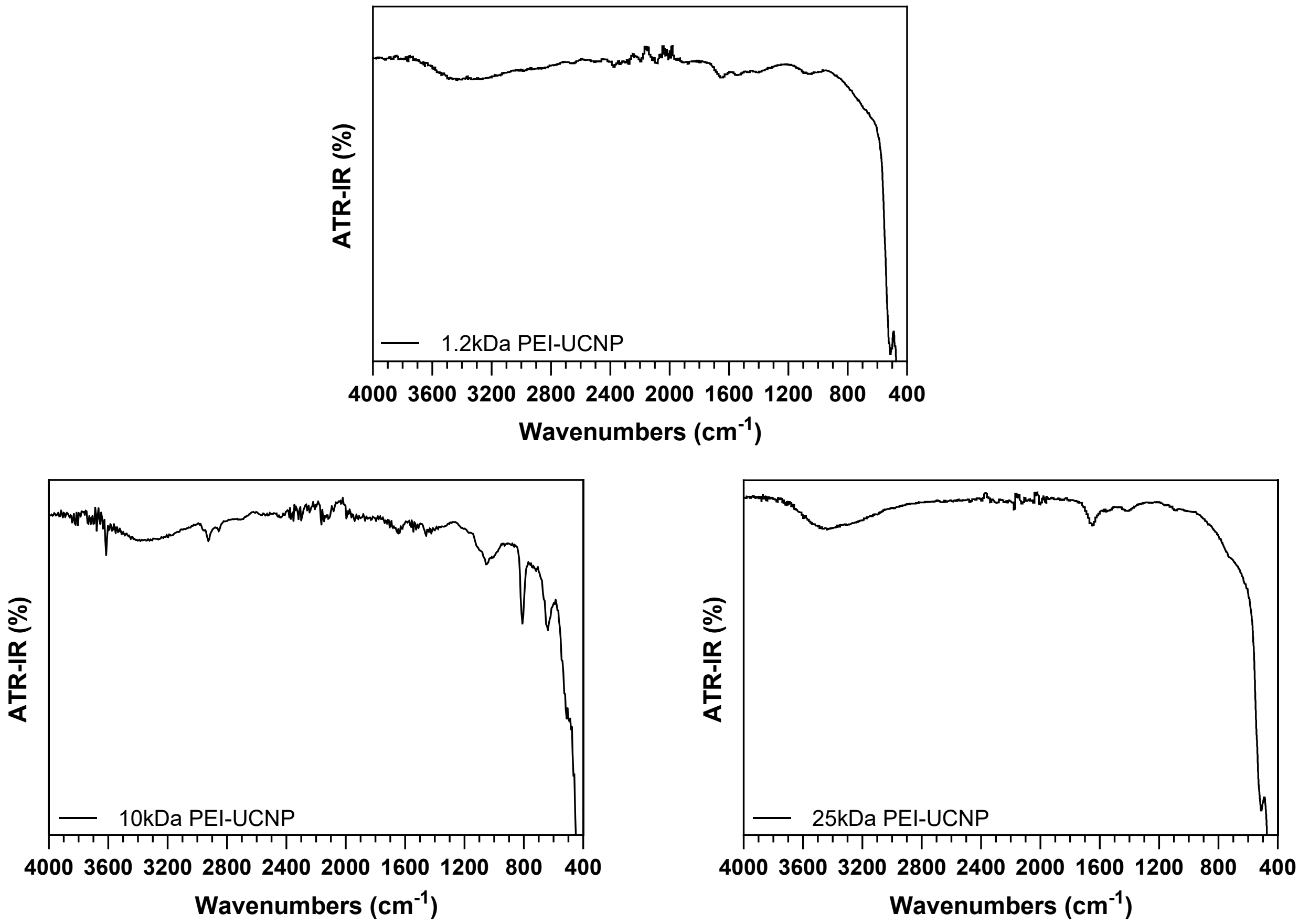

SI Figure 3. **FTIR-ATR of PEI-UCNPs.** In the FTIR-ATR spectrum of the PEI-UCNPs an absorption band is observed around 600 - 400 cm^{-1}, corresponding to the characteristic stretching frequencies of Y-O (metal-oxygen) bond as reported in the literature. A broad absorption band is observed between 3800 – 3000 cm^{-1} attributed to the N-H stretching, together with a band in the range of 1700 - 1500 cm^{-1} , assigned to the primary amine N-H bending.

Primary Amine Presence on UCNP Surface using Ninhydrin Assay

Solution A was prepared by adding 16.5 mg of potassium cyanide into 25 mL milli-Q water. 1 mL of this solution was then added to 49 mL pyridine. Solution B was prepared by adding 1 g ninhydrin hydrate into 20 mL of n-butanol. Solution C was prepared by adding 40 g phenol into 20 mL n-butanol. Stock solutions of PEI-UCNPS (mw 1.2, 10 and 25 kDa) at concentrations of 10 mg/mL. 50 uL of PEI-UCNP stock was pipetted into a 1.5 mL Eppendorf tube. 50 µL of Solution A and C were added followed by 100 µL of Solution B. The tube was closed and placed in a preheated heating block, incubated for 10 minutes at 100 °C. A 10 µL aliquot of the suspension was transferred to a 96 well-plate along with 90 µL of 80% aqueous ethanol solution and the absorbance was measured at 570 nm. For calibration, known concentrations

of butylamine were prepared by serial dilution and was measured by the same protocol as described above to give a standard curve.

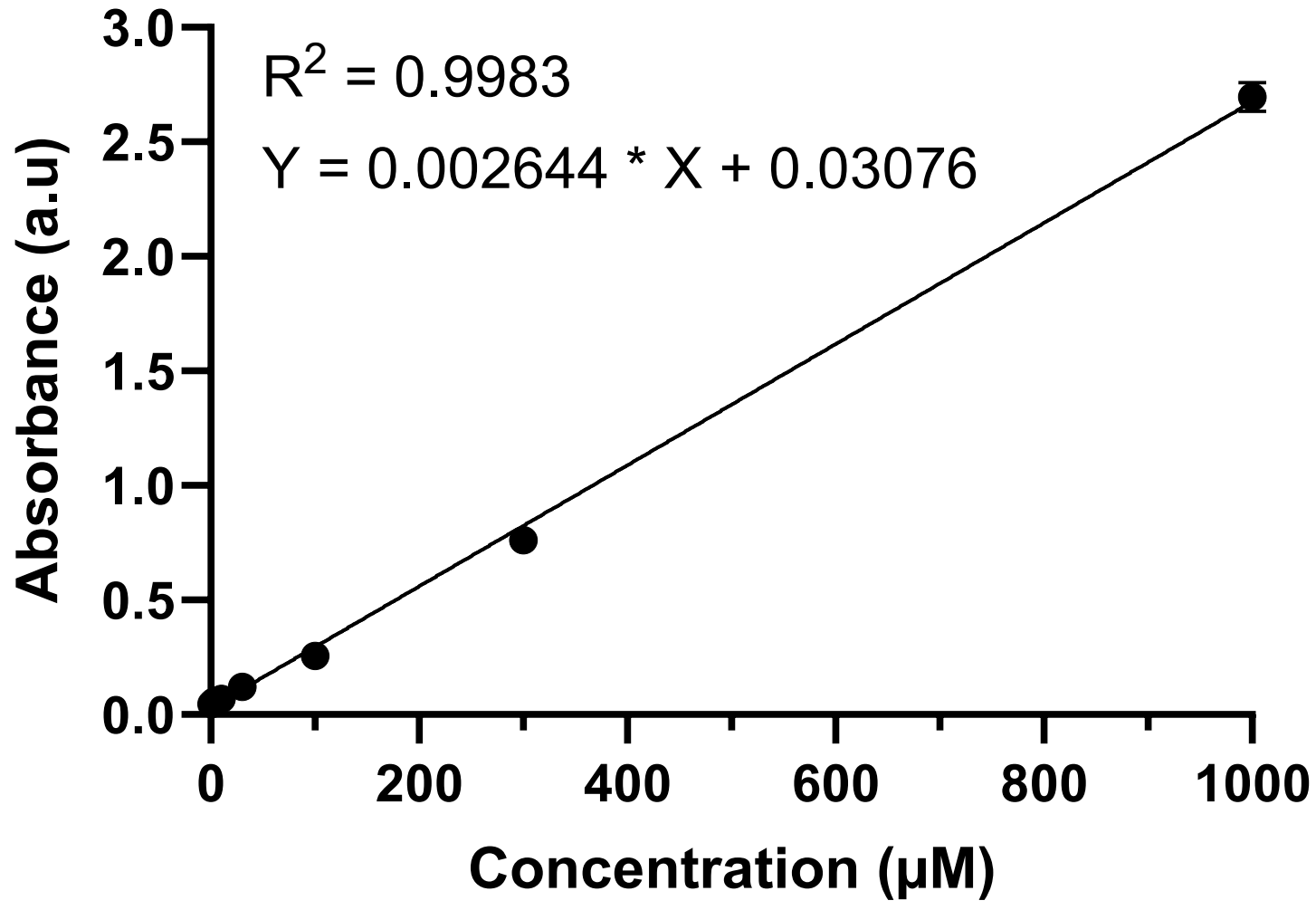

SI Figure 4. **Calibration standard curve for concentration determination.** To determine unknown amine concentrations, a standard calibration curve was constructed using known concentrations of butylamine ranging from 0 to 1000 μM (Figure 4). Absorbance values were plotted against concentration to obtain a linear regression equation of Y= 0.002644 * X + 0.03076, where Y represents absorbance (a.u.) and X represents concentration (μM). The fit showed high linearity with a coefficient of determination (R^2) of 0.9983. Error bars indicate the standard deviation of independent replicates.

SI Table 2. Primary amine presence on UCNPs via Ninhydrin Assay.

Sample	Abs	Concentration (μM)	Average Calculated NH_2 Conc	Primary Amine Presence on UCNPs
**1.2 kDa PEI-UCNP**	0.156	47.370	56.03 ± 9.27 μM	**0.280 mM** per mg of UCNP
	0.199	63.760		
	0.181	56.952		
**10 kDa PEI-UCNP**	0.273	91.498	87.80 ± 8.58 μM	**0.439 mM** per mg of UCNP
	0.248	82.168		
	0.268	89.733		
**25 kDa PEI-UCNP**	0.244	80.529	78.42 ± 6.27 μM	**0.392 mM** per mg of UCNP
	0.230	75.486		
	0.240	79.268		

SI Section 4

ICP-MS results

SI Table 3. ICP-MS data for PEI-UCNPs. Values are reported as mean ± standard deviation from triplicate measurements (n = 3).

	Amount per sample (mg/L)		
**Element**	1.2 kDa	10 kDa	25 kDa
**Na^{+}**	6.6 ± 0.7	6.8 ± 0.8	6.4 ± 0.5
**Y^{3+}**	36.5 ± 0.8	36.4 ± 0.3	36.5 ± 1.7
**Er^{3+}**	1.7 ± 0.2	1.8 ± 0.1	1.7 ± 0.3
**Yb^{3+}**	17.5 ± 1.8	18.9 ± 0.2	18.2 ± 1.8

SI Table 4. Elemental ratio for $NaYF_4$:Yb,Er PEI-UCNPs – normalized to Y^{3+}.

Sample	Y^{3+}	Yb^{3+}	Er^{3+}	Yb^{3+}:Er^{3+} Ratio
**1.2 kDa**	1.000	0.374	0.036	10.3
**10 kDa**	1.000	0.405	0.039	10.5
**25 kDa**	1.000	0.389	0.036	10.7

SI Section 5

UCL and LT analysis of PEI-UCNPs

SI Table 5. Summarised PEI-UCNP LTs in D_2O vs H_2O @ 540 nm.

Polymer chain length	LT (D_2O)(μs)	LT (H_2O)(μs)	Lifetime reduction H_2O vs D_2O (%)
**1.2**	61.8 ± 2.8	46.5 ± 2.9	**24.8**
**10**	48.6 ± 5.3	35.1 ± 3	**27.8**
**25**	51.8 ± 1.9	38.2 ± 1.7	**26.3**

SI Table 6. Summarised PEI-UCNP LTs in D_2O vs H_2O @ 650 nm.

Polymer chain length	LT (D_2O)(μs)	LT (H_2O)(μs)	Lifetime reduction H_2O vs D_2O (%)
**1.2**	161.8 ± 6.3	152.7 ± 2.1	**5.6**
**10**	125.2 ± 2.7	117.0 ± 0.1	**6.6**
**25**	129.1 ± 2.4	113.9 ± 6.8	**11.8**

SI Section 6

FRET efficiency of UCNPs and AF647 in H_2O.

SI Table 7. UCL LTs at 650 nm and associated estimates of FRET efficiency of PEI-UCNPs@AF647 in DI H_2O.

PEI MW (kDa)	LT UCNPs (µs)	Initial AF647 concentration (µM)	LT dye loaded UCNPs (µs)	LT reduction vs no AF647 (%)
**1.2**	152.1 ± 3.8	5	146.5 ± 0.9	**3**
**10**	118.3 ± 11.6	5	112.1 ± 8.6	**5**
**25**	117.5 ± 8.7	5	109.3 ± 6.7	**7**
**1.2**	152.1 ± 3.8	10	141.0 ± 2.0	**7**
**10**	118.3 ± 11.6	10	108.8 ± 10.0	**8**
**25**	117.5 ± 8.7	10	104.5 ± 7.4	**11**

SI Table 8. UCL intensity and associated estimates of FRET efficiency of UCNPs@AF647 in DI H_2O. Comparison of integrated intensity (636-700 nm) for PEI-UCNPs and PEI-UCNPs@AF647 in H_2O.

PEI MW (kDa)	Integrated intensity* (UCNPs)	Initial AF647 concentration (µM)	Integrated intensity* (a.u) (UCNPs@AF647)	Intensity reduction vs no AF647 (%)
**1.2**	328.89	5	141.06	**57**
**10**	360.78	5	153.93	**57**
**25**	376.79	5	119.15	**68**
**1.2**	328.89	10	119.16	**64**
**10**	360.78	10	136.24	**62**
**25**	376.79	10	164.29	**56**

* 636-700 nm.

SI Table 9. Comparison of peak intensity @652 nm of PEI-UCNPs vs PEI-UCNPs@AF647 in H_2O.

PEI MW (kDa)	AF647 concentration (μM)	Peak intensity (a.u) @652 nm (UCNPs)	Peak intensity (a.u) @652 nm (UCNP@AF647)	% Quenching
**1.2**	5	11.75	3.39	71
**10**	5	12.61	3.73	70
**25**	5	13.58	4.10	70
**1.2**	10	11.75	2.91	75
**10**	10	12.61	3.76	70
**25**	10	13.80	3.00	78

SI Table 10. Comparison of peak intensity @670 nm of PEI-UCNPs vs PEI-UCNPs@AF647 in H_2O.

PEI MW (kDa)	AF647 concentration (μM)	Peak intensity (a.u) @670 nm (UCNPs)	Peak intensity (a.u) @670 nm (UCNP@AF647)	% Quenching
**1.2**	5	13.39	6.85	49
**10**	5	15.02	8.00	47
**25**	5	15.30	8.30	46
**1.2**	10	13.39	6.63	51
**10**	10	15.02	7.50	50
**25**	10	15.30	6.40	58

SI Section 7

FRET efficiency (UCL method) of UCNPs and AF647 in D_2O.

SI Table 11. UCL LTs and associated estimates of FRET efficiency of PEI-UCNPs@AF647 in D_2O.

PEI MW (kDa)	AF647 concentration (μM)	τ UCNPs	τ dye loaded UCNPs	LT reduction vs no AF647 (%)
**1.2**	5	169.1 ± 3.9	156.9 ± 6.1	**7**
**10**	5	124.7 ± 9.3	117.0 ± 9.7	**6**
**25**	5	129.6 ± 6.0	117.7 ± 7.0	**9**
**1.2**	10	169.1 ± 3.9	147.4 ± 1.6	**13**
**10**	10	124.7 ± 9.3	111.5 ± 10.7	**11**
**25**	10	129.6 ± 6.0	109.4 ± 9.0	**16**

SI Table 12. UCL intensity and associated estimates of FRET efficiency of PEI-UCNPs@AF647 in D_2O. Comparison of integrated intensity (636-700 nm) for PEI-UCNPs and PEI-UCNPs@AF647 in D_2O.

PEI MW (kDa)	AF647 concentration (μM)	Integrated intensity (a.u) between 636-700 nm (UCNPs)	Integrated intensity (a.u) between 636-700 nm (UCNPs@AF647)	Intensity reduction vs no AF647 (%)
**1.2**	5	99.81	15.00	**85**
**10**	5	134.85	35.46	**73**
**25**	5	114.27	20.79	**82**
**1.2**	10	99.81	24.41	**76**
**10**	10	134.85	37.86	**72**
**25**	10	114.27	22.65	**80**

SI Table 13. Comparison of emission intensity @652 nm for PEI-UCNPs vs PEI-UCNPs@AF647 in D_2O.

PEI MW (kDa)	AF647 concentration (μM)	Peak intensity (a.u) @652 nm (UCNPs)	Peak intensity (a.u) @652 nm (UCNP@AF647)	% Quenching
**1.2**	5	3.57	0.41	88.52
**10**	5	4.85	0.92	81.03
**25**	5	4.01	0.57	85.79
**1.2**	10	3.57	0.65	81.79
**10**	10	4.85	0.88	81.86
**25**	10	4.01	0.60	85.04

SI Table 14. **Comparison of emission intensity @670 nm for UCNP vs UCNP@AF647 peak in D_2O.**

PEI MW (kDa)	AF647 concentration (μM)	Peak intensity (a.u) @670 nm (UCNPs)	Peak intensity (a.u) @670 nm (UCNP@AF647)	% Quenching
**1.2**	5	4.13	0.73	82.30
**10**	5	5.53	1.74	68.54
**25**	5	4.74	1.03	78.27
**1.2**	10	4.13	1.30	68.52
**10**	10	5.53	2.21	60.04
**25**	10	4.74	1.18	75.11

SI Section 8

LT data fits for UCNPs in H_2O and D_2O.

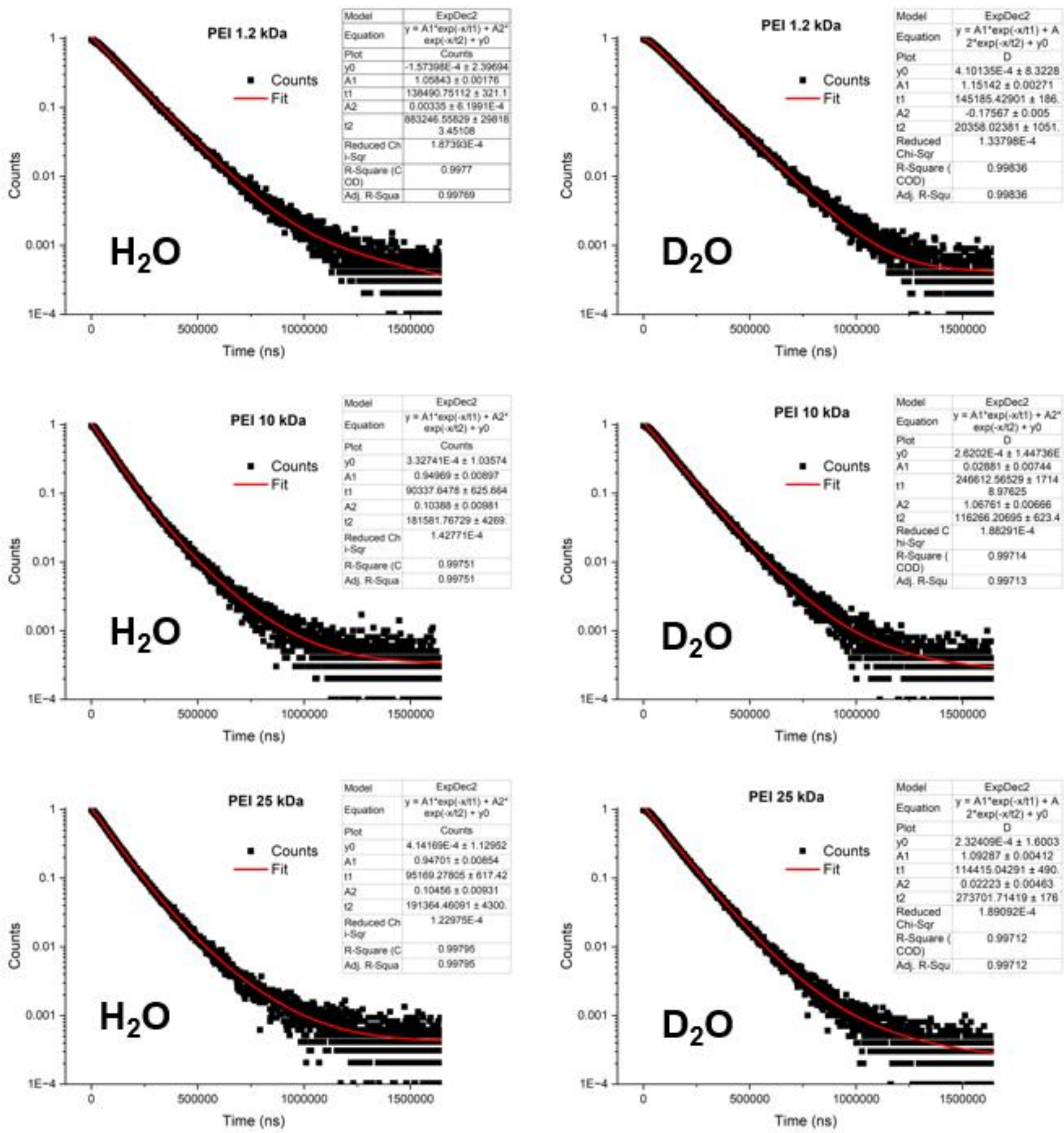

SI Figure 5. UCNPs (10 mg/mL) in H_2O vs D_2O fitted LT data using ExpDec2 function on Origin2025. Top row: 1.2 kDa PEI UCNPs. Middle row: 10 kDa PEI UCNPs. Bottom row: 25 kDa PEI UCNPs. Left column: DI H_2O. Right column, D_2O.

PEI UCNPs@5 µM AF647 in H_2O and D_2O:

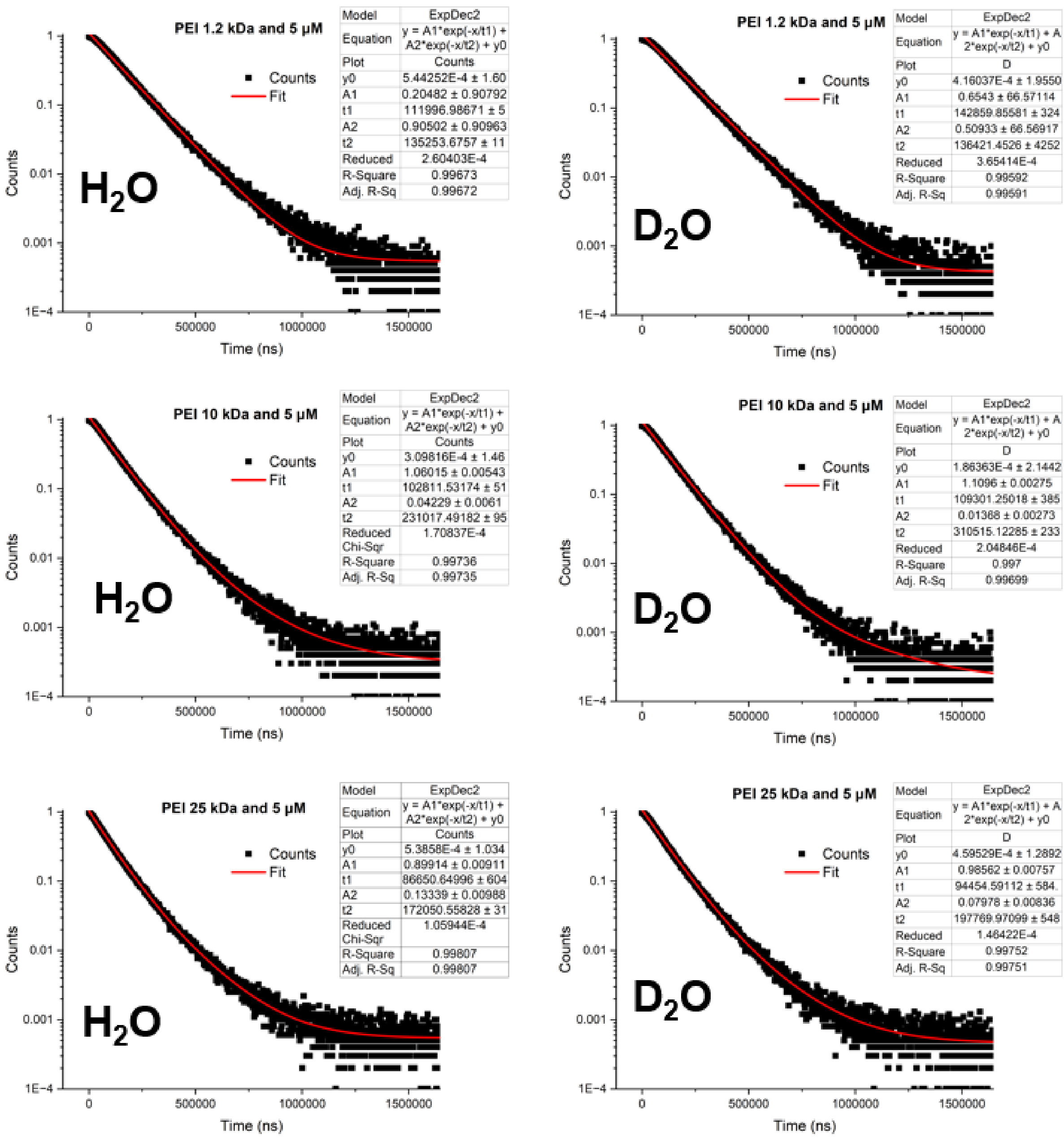

SI Figure 6. UCNPs (10 mg/mL)@ AF647 in H2O vs D2O fitted LT data using ExpDec2 function on Origin2025. Top row: 1.2 kDa PEI UCNPs. Middle row: 10 kDa PEI UCNPs. Bottom row: 25 kDa PEI UCNPs. Left column: DI H_2O. Right column, D_2O.

PEI UCNPs@10 μM AF647 in H_2O and D_2O:

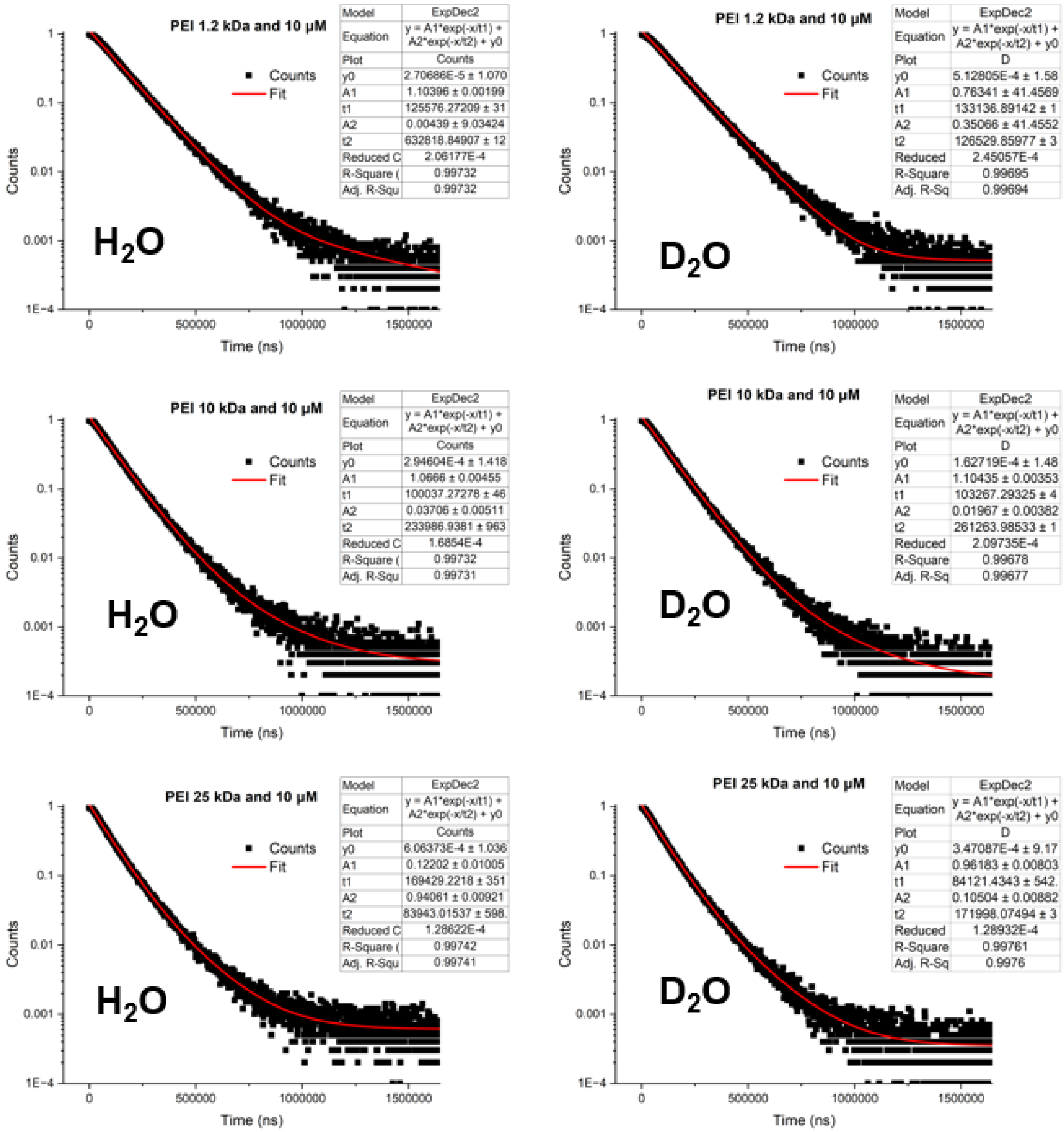

SI Figure 7. UCNPs (10 mg/mL)@ AF647(10 μM initial concentration) in H_2O vs D_2O fitted LT data using ExpDec2 function on Origin2025. Top row: 1.2 kDa PEI UCNPs. Middle row: 10 kDa PEI UCNPs. Bottom row: 25 kDa PEI UCNPs. Left column: DI H_2O. Right column, D_2O.

SI Section 9

Statistical analysis

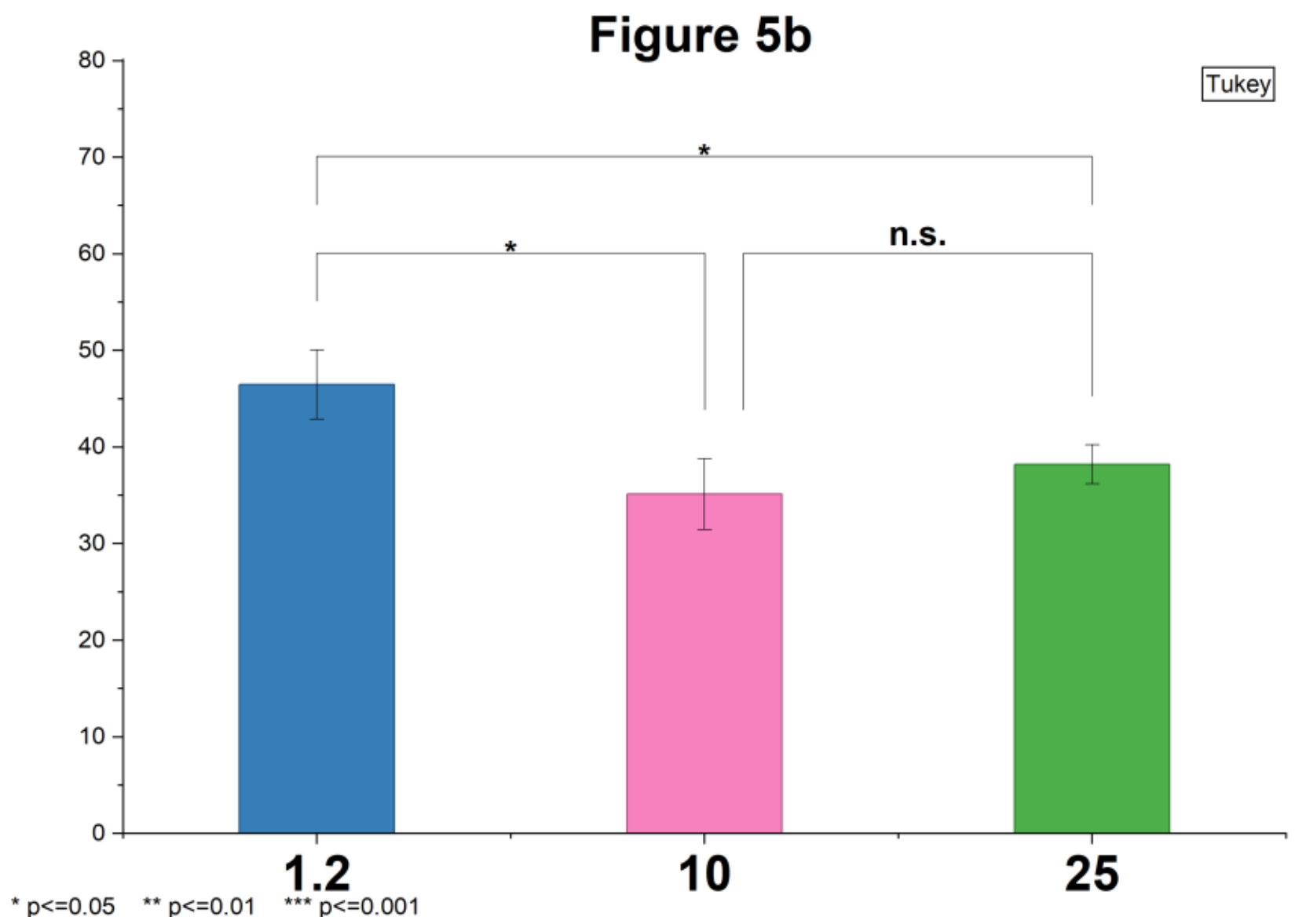

SI Figure 8. Statistical significance (Figure 5b) was assessed using ordinary one-way ANOVA followed by Tukey's multiple-comparisons test. Adjusted p-values were: 1.2 vs 10 kDa, p=0.011; 1.2 vs 25 kDa, p = 0.044; and 10 vs 25 kDa, p= 0.503. * p<0.05, ** p< 0.01, *** p< 0.001. Comparisons without an asterisk were not statistically significant (p ≥ 0.05).

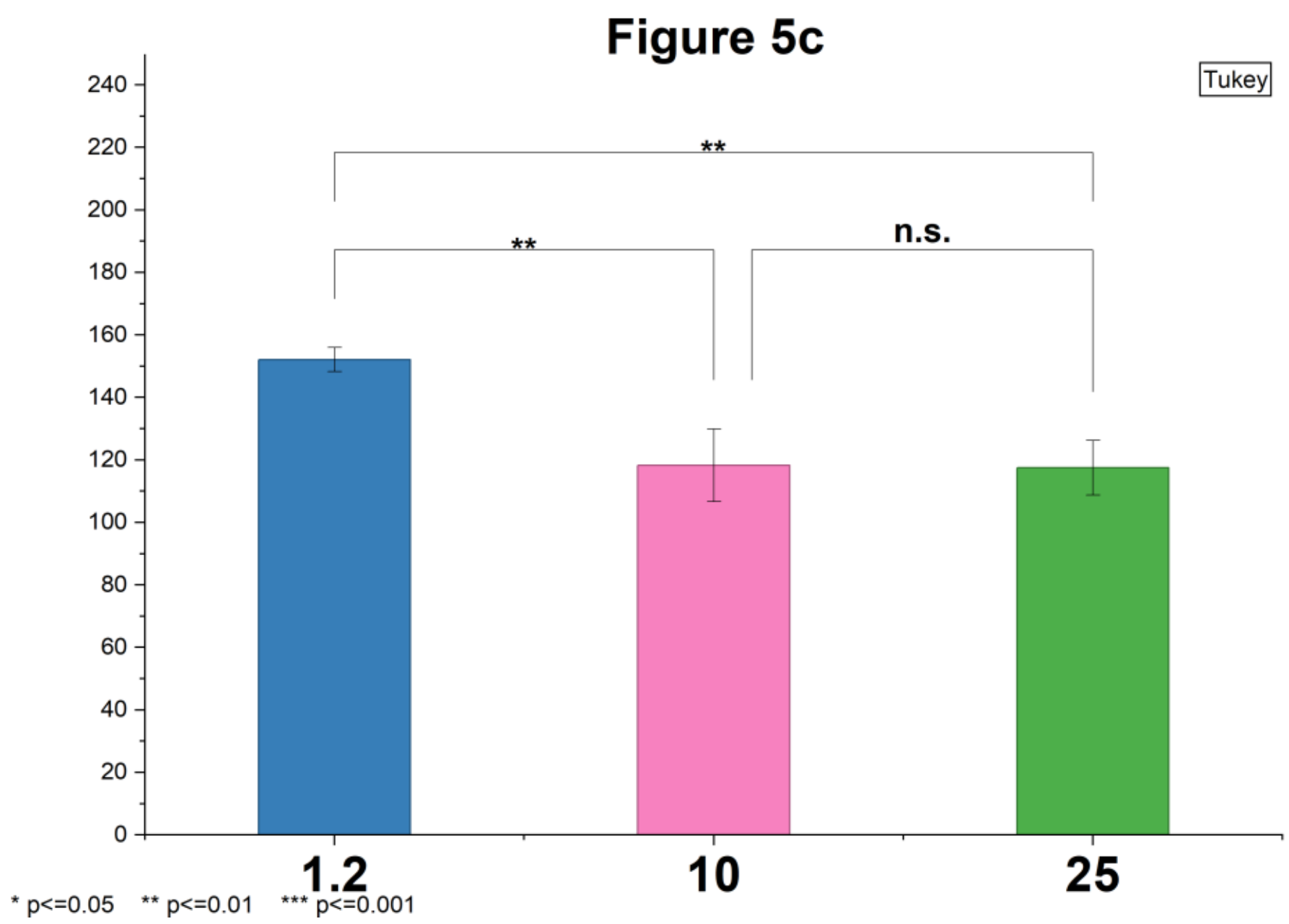

SI Figure 9. Statistical significance (Figure 5c) was assessed using ordinary one-way ANOVA followed by Tukey's multiple-comparisons test. Adjusted p-values were: 1.2 vs 10 kDa, p=0.007; 1.2 vs 25 kDa, p = 0.007; and 10 vs 25 kDa, p= 0.994. * p<0.05, ** p< 0.01, *** p< 0.001. Comparisons without an asterisk were not statistically significant (p ≥ 0.05).

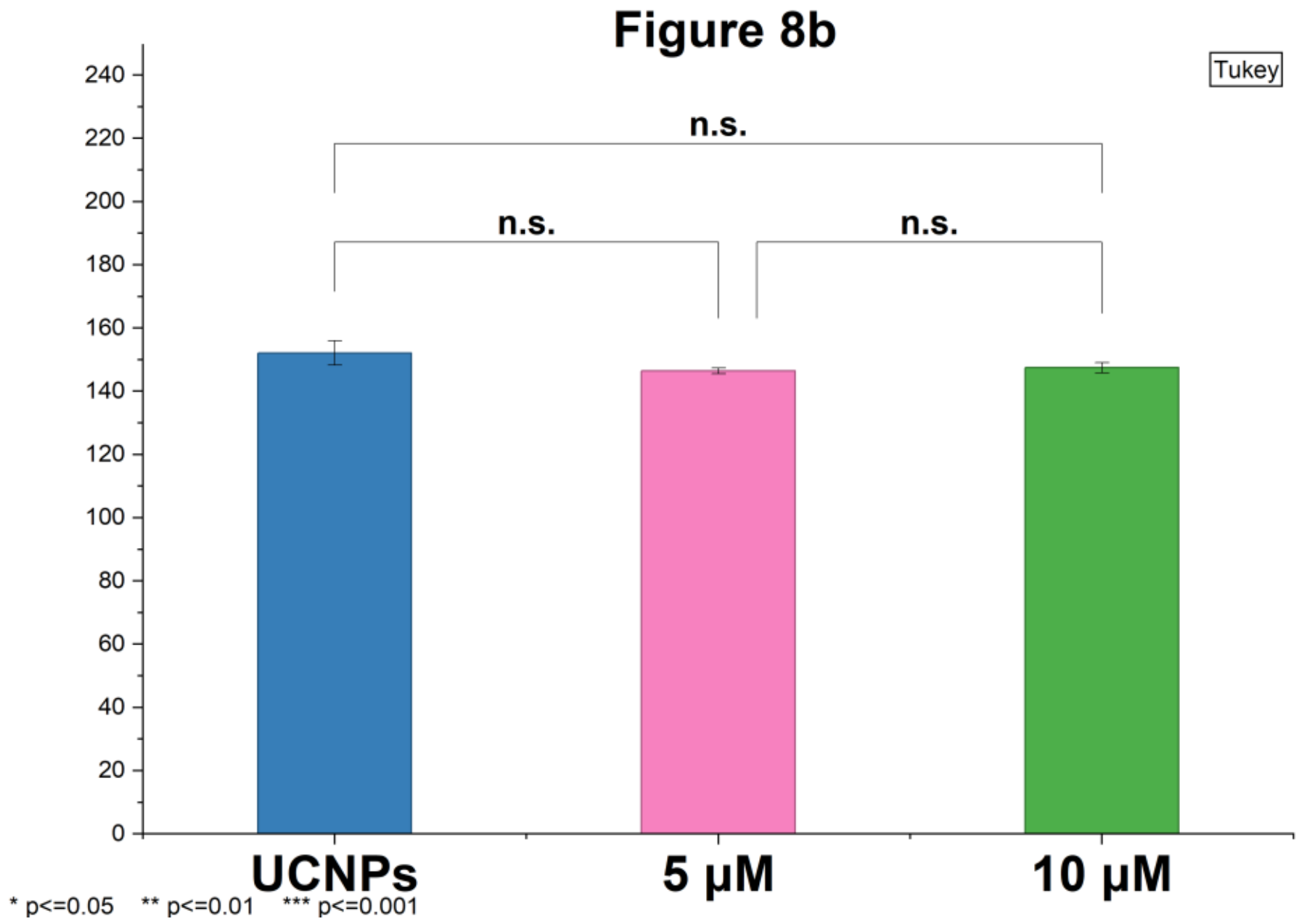

SI Figure 10. Statistical significance of the LT measurements of 1.2 kDa UCNPs and AF647 (Figure 8b) was assessed using ordinary one-way ANOVA followed by Tukey's multiple-comparisons test. Tukey-adjusted p-values were 0.07018 for UCNPs alone versus 5 µM AF647, 0.12806 for UCNPs alone versus 10 µM AF647, and 0.88593 for 5 versus 10 µM AF647. No comparisons were statistically significant ($p \geq 0.05$).

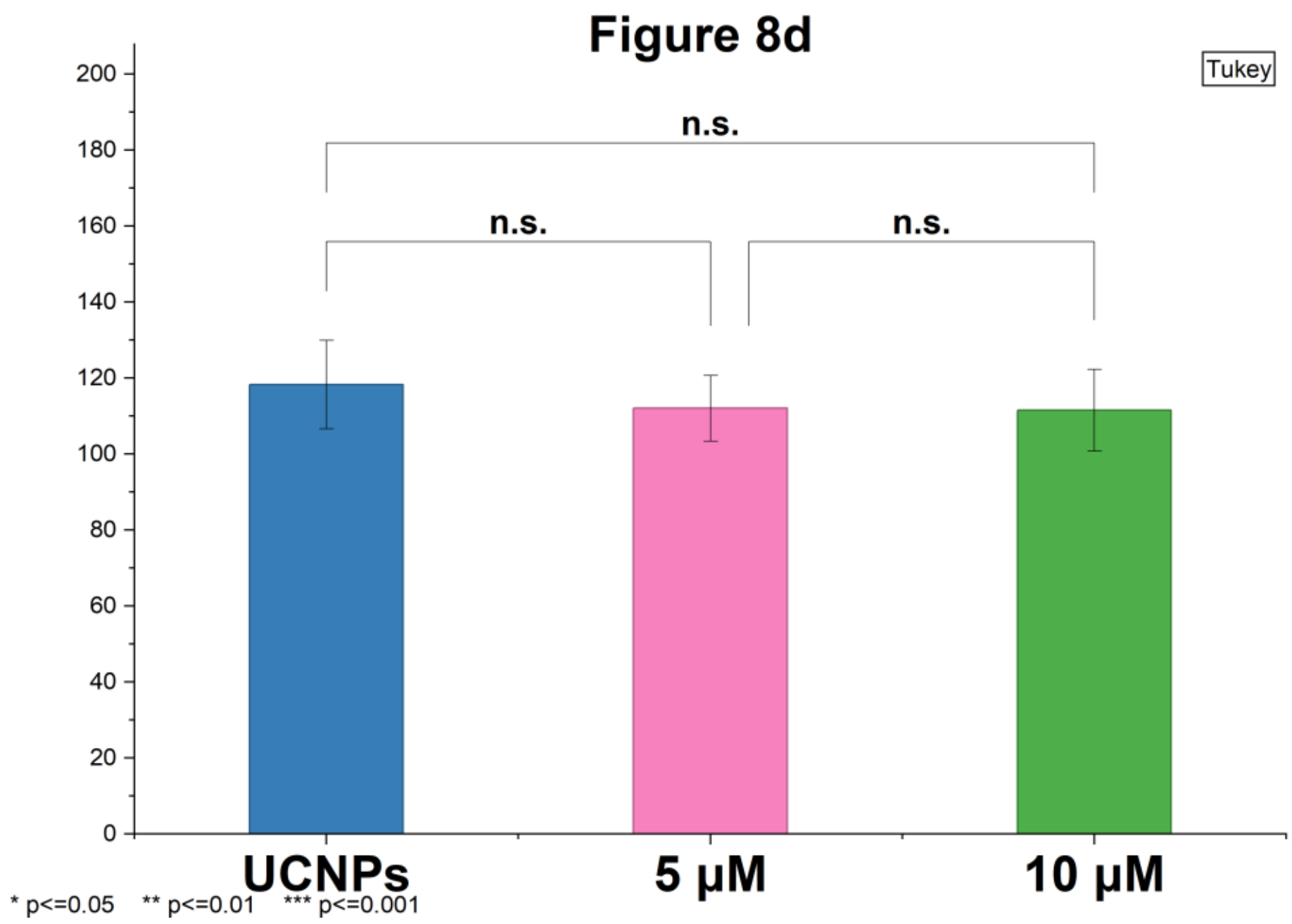

SI Figure 11. Statistical significance of the LT measurements of 10 kDa UCNPs and AF647 (Figure 8d) was assessed using ordinary one-way ANOVA followed by Tukey's multiple-comparisons test. Tukey-adjusted p-values were 0.75742 for UCNPs alone versus 5 µM AF647, 0.72238 for UCNPs alone versus 10 µM AF647, and 0.99783 for 5 versus 10 µM AF647. No comparisons were statistically significant ($p \geq 0.05$).

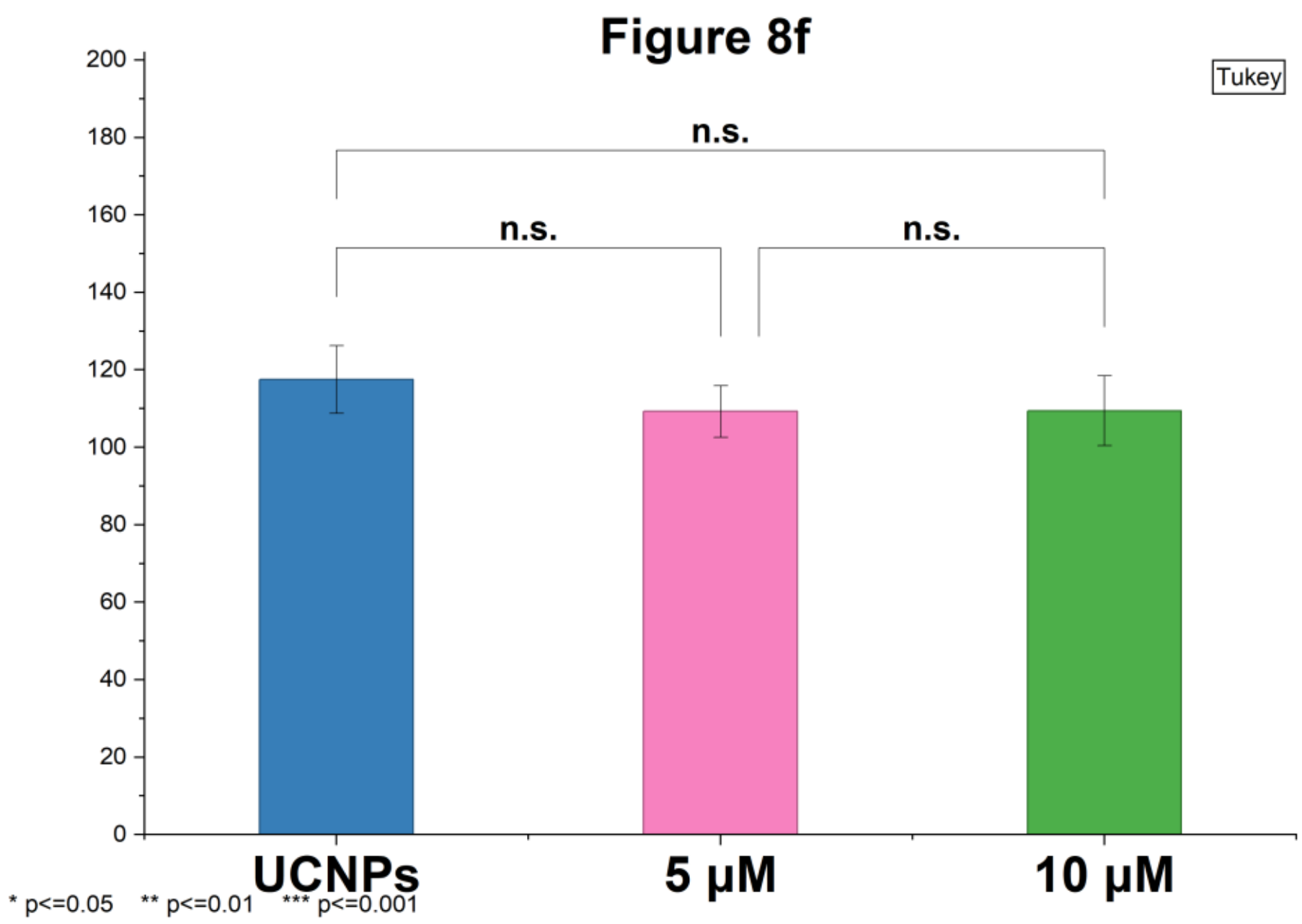

SI Figure 12. Statistical significance of the LT measurements of 25 kDa UCNPs and AF647 (Figure 8d) was assessed using ordinary one-way ANOVA followed by Tukey's multiple-comparisons test. Tukey-adjusted p-values were 0.47895 for UCNPs alone versus 5 µM AF647, 0.49134 for UCNPs alone versus 10 µM AF647, and 0.99967 for 5 versus 10 µM AF647. No comparisons were statistically significant ($p \geq 0.05$).

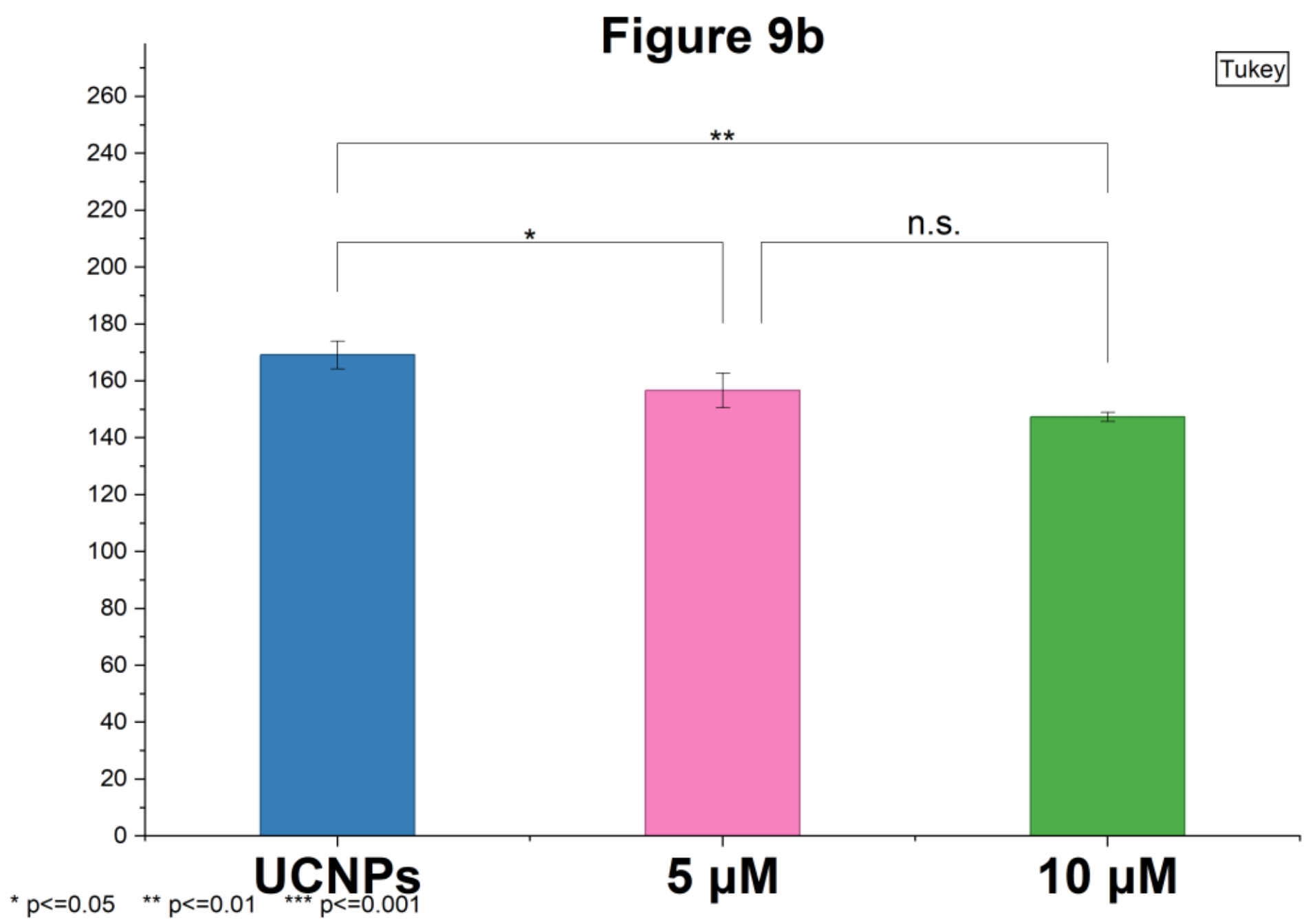

SI Figure 13. Statistical significance of the LT measurements of 1.2 kDa UCNPs and AF647 in D_2O (Figure 9b) was assessed using ordinary one-way ANOVA followed by Tukey's multiple-comparisons test. Tukey-adjusted p-values were 0.037 for UCNPs alone versus 5 µM AF647, 0.0028 for UCNPs alone versus 10 µM AF647, and 0.10 for 5 versus 10 µM AF647. Only

statistically significant comparisons are indicated; unmarked comparisons were not significant ($p \geq 0.05$). *$p < 0.05$ and **$p < 0.01$.

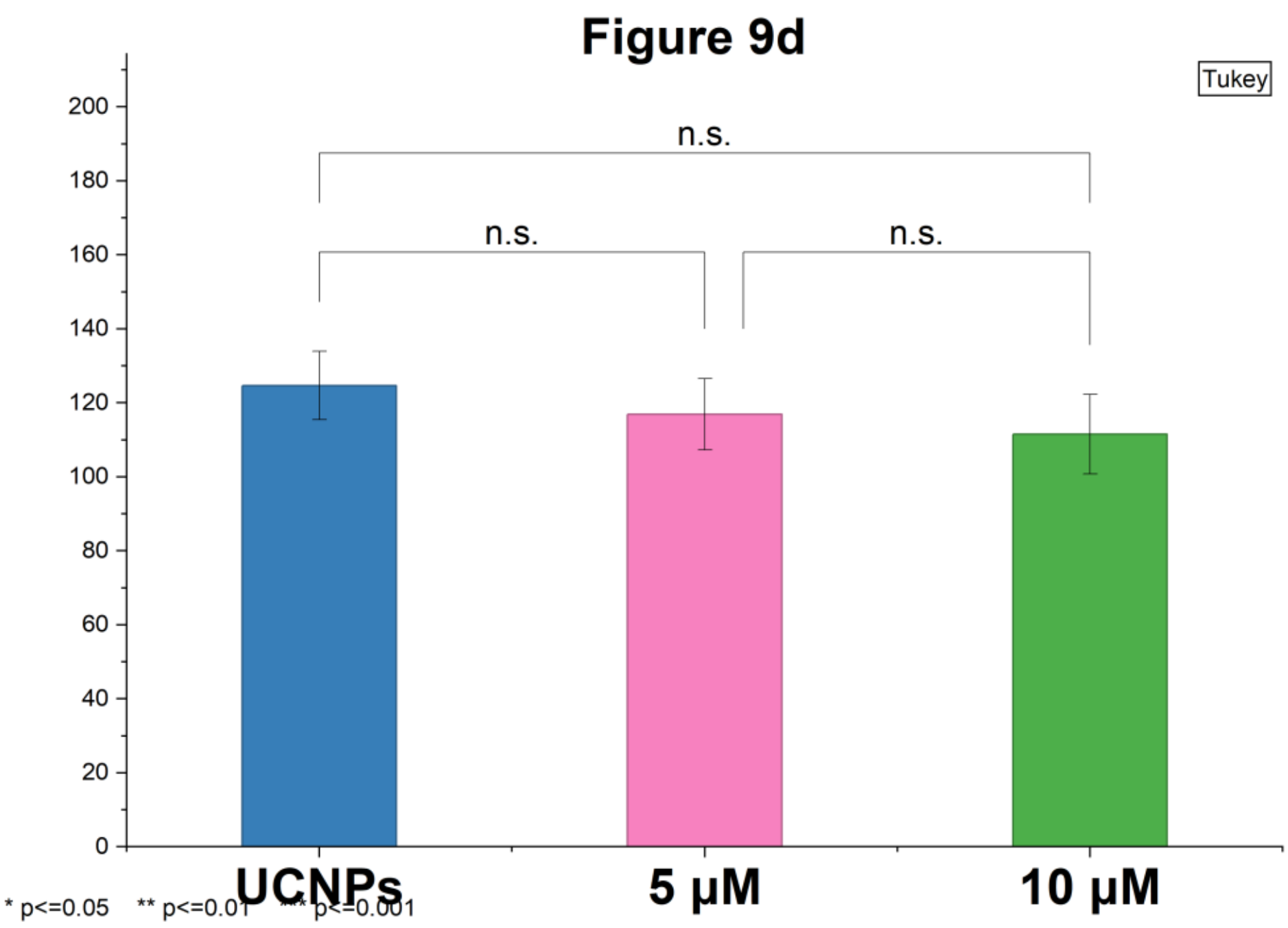

SI Figure 14. Statistical significance of the LT measurements of 10 kDa UCNPs and AF647 in D_2O (Figure 9d) was assessed using ordinary one-way ANOVA followed by Tukey's multiple-comparisons test. Tukey-adjusted p-values were 0.63 for UCNPs alone versus 5 µM AF647, 0.31 for UCNPs alone versus 10 µM AF647, and 0.79 for 5 versus 10 µM AF647. No comparisons were statistically significant ($p \geq 0.05$).

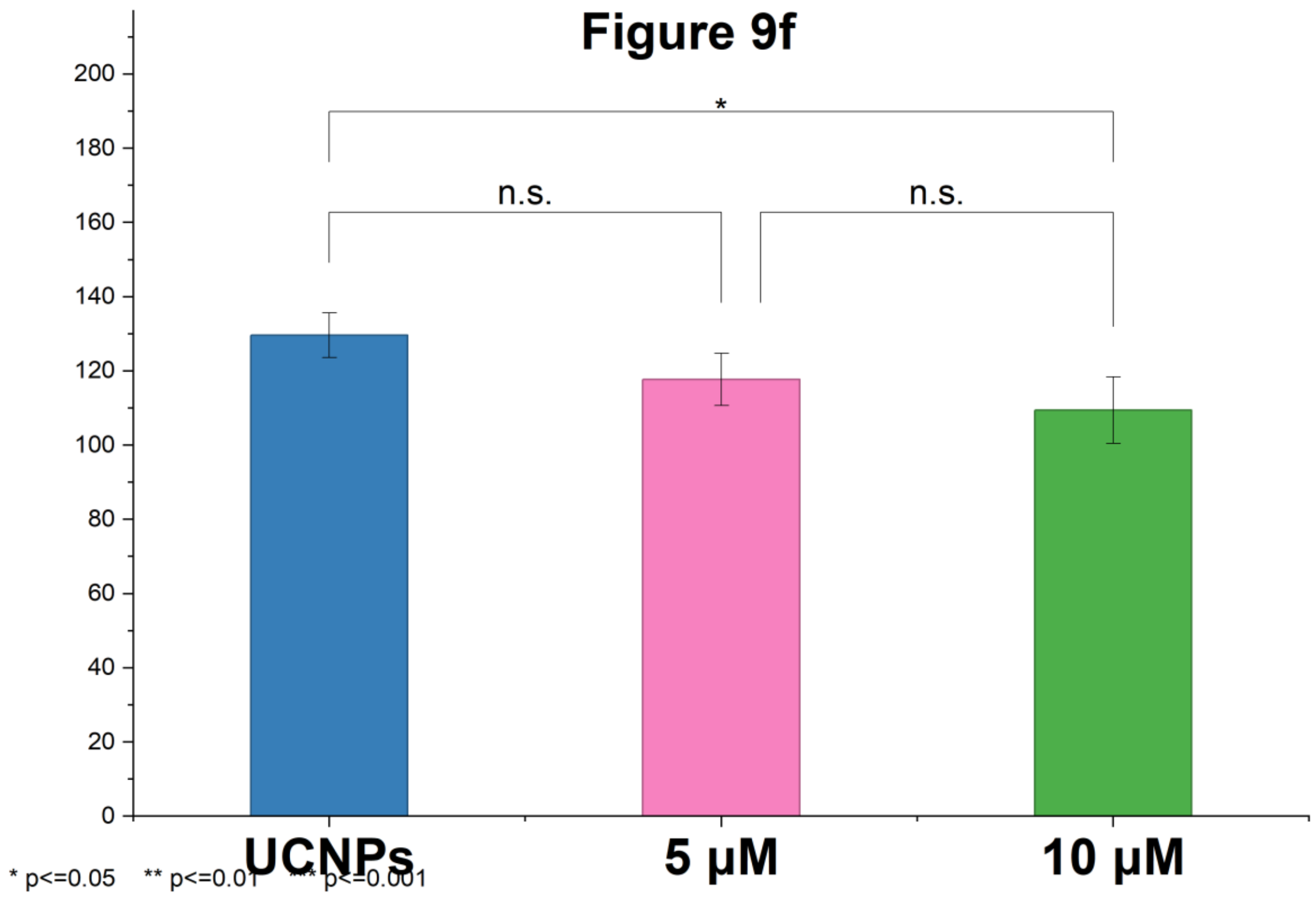

SI Figure 15. Statistical significance of the LT measurements of 25 kDa UCNPs and AF647 in D_2O (Figure 9d) was assessed using ordinary one-way ANOVA followed by Tukey's multiple-comparisons test. Tukey-adjusted p-values were 0.20 for UCNPs alone versus 5 µM AF647, 0.036 for UCNPs alone versus 10 µM AF647, and 0.41 for 5 versus 10 µM AF647. Only statistically significant comparisons are indicated; unmarked comparisons were not significant ($p \geq 0.05$). *$p < 0.05$.

SI Section 10

Calculation of spectral overlap, Förster radius and apparent donor-acceptor distance

FRET calculations were performed to estimate the spectral overlap between the PEI-UCNP donor and AF647 acceptor, the corresponding Förster radius (R_0) and the apparent donor-acceptor distance (R). Calculations were performed separately for UCNPs prepared using 1.2, 10 and 25 kDa PEI in H_2O and D_2O. All calculations were performed using Origin2026.

Conversion of AF647 absorbance to molar extinction coefficient

The absorption spectrum of 5 μM AF647 was measured between 500 and 800 nm using a 1 cm path-length cuvette. A baseline correction was first applied using the mean absorbance between 750 and 800 nm which was:

$$A_{baseline} = -0.009813$$

The corrected absorbance was therefore calculated as:

$$A_{corrected} = A_{measured} + 0.009813$$

Any negative abs values remaining following baseline correction were set to 0.

The corrected AF647 absorbance spectrum was then converted into molar extinction coefficient, ε, using the beer-lambert relationship:

$$A = \varepsilon c l$$

Therefore:

$$\varepsilon = \frac{A}{cl} = \frac{A_{corrected}}{5x10^{-6}} = 1.61 \times 10^{5}\ M^{-1}cm^{-1}$$

Preparation of the UCNP donor emission spectra

The donor-only UCNP emission spectra were used for overlap calculations. Calculations were performed over 626-706 nm, corresponding to the spectral region relevant to overlap between red Er^{3+} UCNP emission and AF647 absorption. Separate donor emission spectra were used for 1.2, 10 and 25 kDa PEI-UCNPs in H_2O and 1.2, 10 and 25 kDa UCNPs in D_2O. The integrated area of each donor emission spectrum between 626 and 706 nm was first determined and each emission spectrum was then area-normalised. This step was used to ensure the overlap calculation depended on the spectral distribution of donor emission, rather than its absolute emission intensity.

Matching the AF647 absorption and UCNP emission wavelengths

The AF647 molar extinction coefficient spectrum and UCNP emission spectra were collected using different wavelength points. The AF647 values were therefore linearly interpolated onto the UCNP wavelength values between 626 and 706 nm. This produced, for every UCNP

wavelength λ, a corresponding normalised UCNP donor emission, $F_D(\lambda)$, and AF647 molar extinction coefficient, $\varepsilon_A(\lambda)$. These were then used in the overlap calculation.

Calculation of the spectral overlap integral, J

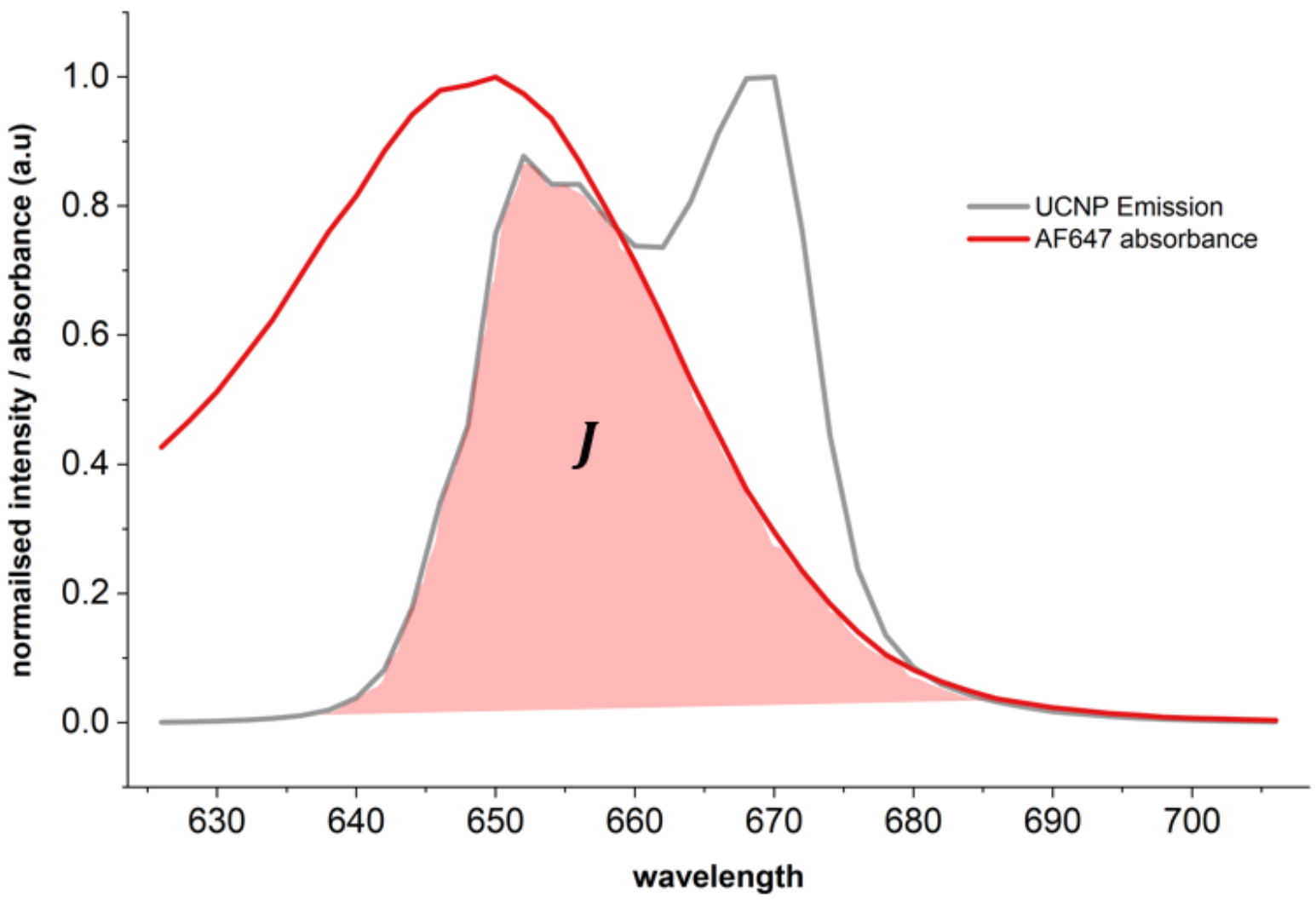

SI Figure 16. **Representative spectral overlap between PEI-UCNP donor emission dn AF647 acceptor absorption.** Normalised emission of 1.2 kDa PEI-UCNPs in H_2O and normalised AF647 absorption are shown with shaded region illustrating the spectral overlap integral (*J*).

The donor-acceptor spectral overlap integral was calculated using:[1]

$$J = \int F_D(\lambda)\varepsilon_A(\lambda)\lambda^4 \mathrm{d}\lambda$$

Where $F_D(\lambda)$ is the normalised UCNP donor emission, $\varepsilon_A(\lambda)$ is the AF647 molar exctinction coefficient and λ is wavelength in nm.

In Origin, the integrand was first calculated at each wavelength using:

$$F_D(\lambda) \times \varepsilon_A \times (\lambda)\lambda^4$$

And this resulting column was integrated between 626 and 706 nm, and the resulting overlap integrals are detailed in Table 15. The similarity between these values indicate that the spectral overlap between UCNP emission and AF647 absorption was essentially unchanged between the PEI molecular weight formulations and between H_2O and D_2O.

SI Table 15. Calculated J integrals of various polymer MW's in H_2O and D_2O.

PEI MW	Solvent	J (M^{-1} cm^{-1} nm^4)
**1.2**	H_2O	1.86406 x10^{16}
**10**	H_2O	1.84610 x10^{16}
**25**	H_2O	1.87044 x10^{16}
**1.2**	D_2O	1.85921 x10^{16}
**10**	D_2O	1.86521 x10^{16}
**25**	D_2O	1.86319 x10^{16}

Calculation of the Forster Radius, R_0

The overlap integral was the use to calculate the Forter radius:[2]

$$R_0\ (nm) = 0.02108\ [\frac{k^2 \phi_D J}{n^4}]^{\frac{1}{6}}$$

Where:

k^2= donor-acceptor orientation factor
ϕ_D= donor quantum yield
J = experimentaly calculated spectral overlap integral
n = refractive index of the medium
An orientation factor of $k^2 = \frac{2}{3}$ was assumed, corresponding to the conventional assumption of randomly orientated donor and acceptor transition dipoles.[3] For H_2O, n =1.333 and for D_2O n = 1.328.

As an appropriate absolute Er^{3+} donor quantum yield could not be directly assigned to the UCNP donor, the calculation was performed using three assumed quantum yields:

$$\phi_D = 0.01, 0.05, 0.10$$

Corresponding to 1%, 5% and 10% respectively. These values therefore provide a range of estimated R_0 values, rather than assuming a single experimentally determined quantum yield. The corresponding J values were substituted into equation $R_0\ (nm)$ and the complete calculated values are shown in Table 16.

SI Table 16. Calculated $R_0\ (nm)$ of various polymer MW's in H_2O and D_2O at $\phi_D = 0.01, 0.05, 0.10$.

PEI MW	Solvent	R_0, QY 1%	R_0, QY 5%	R_0, QY 10%
**1.2kDa**	H_2O	3.89	5.08	5.71
**10 kDa**	H_2O	3.88	5.08	5.7
**25 kDa**	H_2O	3.89	5.09	5.71
**1.2kDa**	D_2O	3.9	5.09	5.72
**10 kDa**	D_2O	3.9	5.1	5.72
**25 kDa**	D_2O	3.9	5.1	5.72

Calculation of intensity-based FRET efficiency

Experimentally determined intensity-based FRET efficiencies were then used to estimate apparent donor-acceptor distances. These were determined from:

$$E = 1 - \frac{I_{DA}}{I_D}$$

Where I_D represents donor emission in the absence of AF647 and I_{DA} represents donor emission in the presence of AF647. The resultant values are quoted as percentages.

Calculation of apparent donor-acceptor distance, R

The apparent donor-acceptor distance was calculated from the experimentally determined FRET efficiency and calculated R_0:

$$R = R_0 \left(\frac{1 - E}{E}\right)^{\frac{1}{6}}$$

For example, for 1.2 kDa PEI-UCNPs + 5 μM AF647 in H_2O, $E = 0.57$. Using the '1%' assumed donor QY:

$$R_0 = 3.89\ nm$$

Therefore:

$$R = 3.89 \left(\frac{1 - 0.57}{0.57}\right)^{\frac{1}{6}}$$

Giving:

$$R = 3.71\ nm$$

The calculation was repeated using the R_0 values obtained for the 5% and 10% assumed quantum yields, giving apparent distances of approximately 4.85 sand 5.84 nm respectively.

The same procedure was the repeated for each PEI MW, AF647 concentration and solvent. Results are detailed in Table 17.

SI Table 17. Calculated R values of various polymer MW's in H_2O and D_2O at $\phi_D = 0.01, 0.05, 0.10$.

PEI MW	Solvent	AF647	E	R, QY 1%	R, QY 5%	R, QY 10%
**1.2**	H_2O	5 μM	57%	3.71	4.85	5.45
**1.2**	H_2O	10 μM	64%	3.53	4.62	5.19
**10**	H_2O	5 μM	57%	3.7	4.85	5.44
**10**	H_2O	10 μM	62%	3.58	4.68	5.25
**25**	H_2O	5 μM	88%	2.79	3.65	4.1
**25**	H_2O	10 μM	56%	3.74	4.89	5.49
**1.2**	D_2O	5 μM	85%	2.92	3.82	4.29
**1.2**	D_2O	10 μM	76%	3.22	4.2	4.72
**10**	D_2O	5 μM	73%	3.3	4.32	4.85
**10**	D_2O	10 μM	72%	3.34	4.36	4.9
**25**	D_2O	5 μM	82%	3.03	3.96	4.45
**25**	D_2O	10 μM	80%	3.1	4.05	4.55

SI Section 11

MALDI-MS data for PEI polymers used in synthesis

3.5.1 1.2 kDa PEI

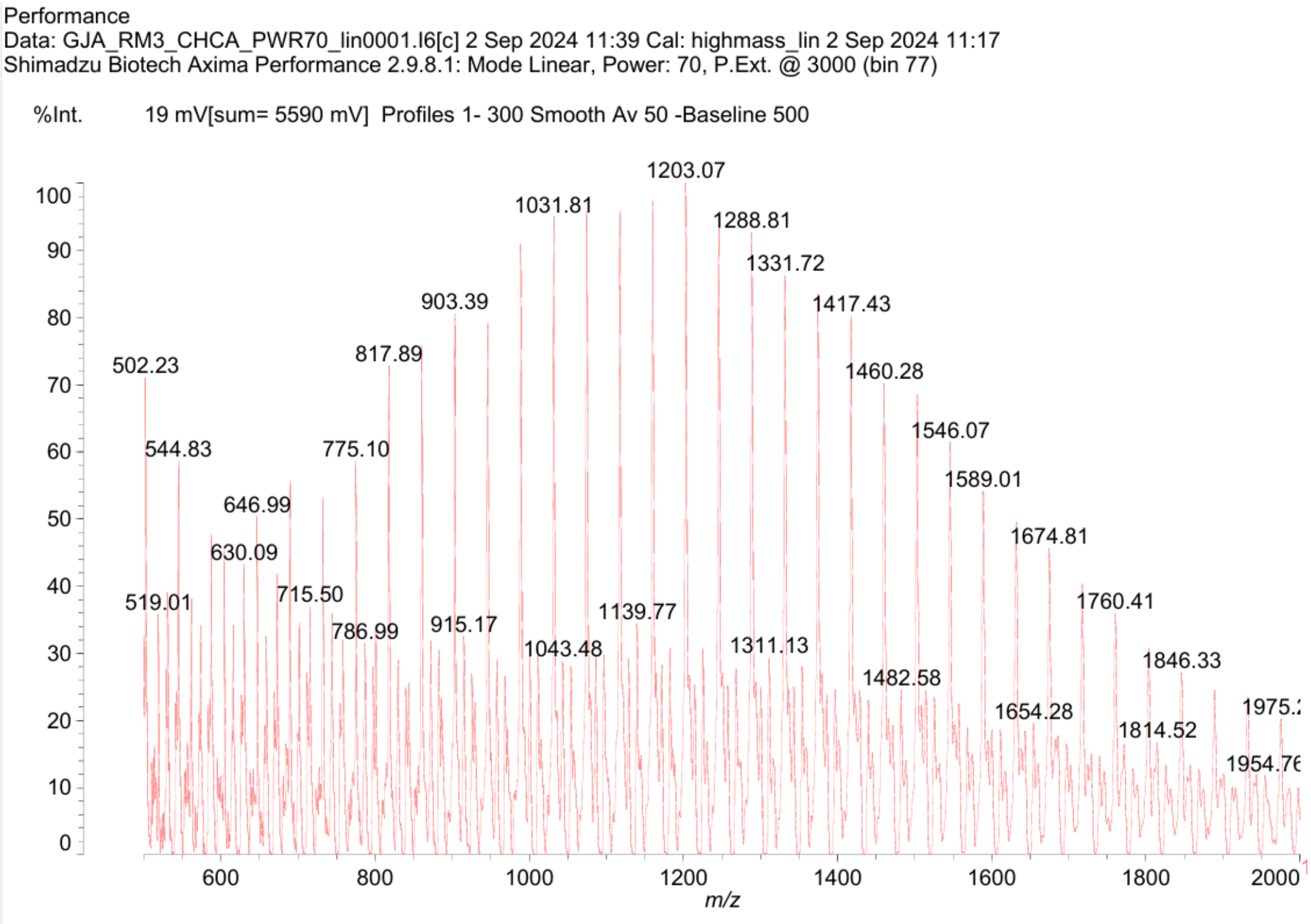

SI Figure 17. MALDI-MS of PEI 1.2 kDa (500-2000 m/z).

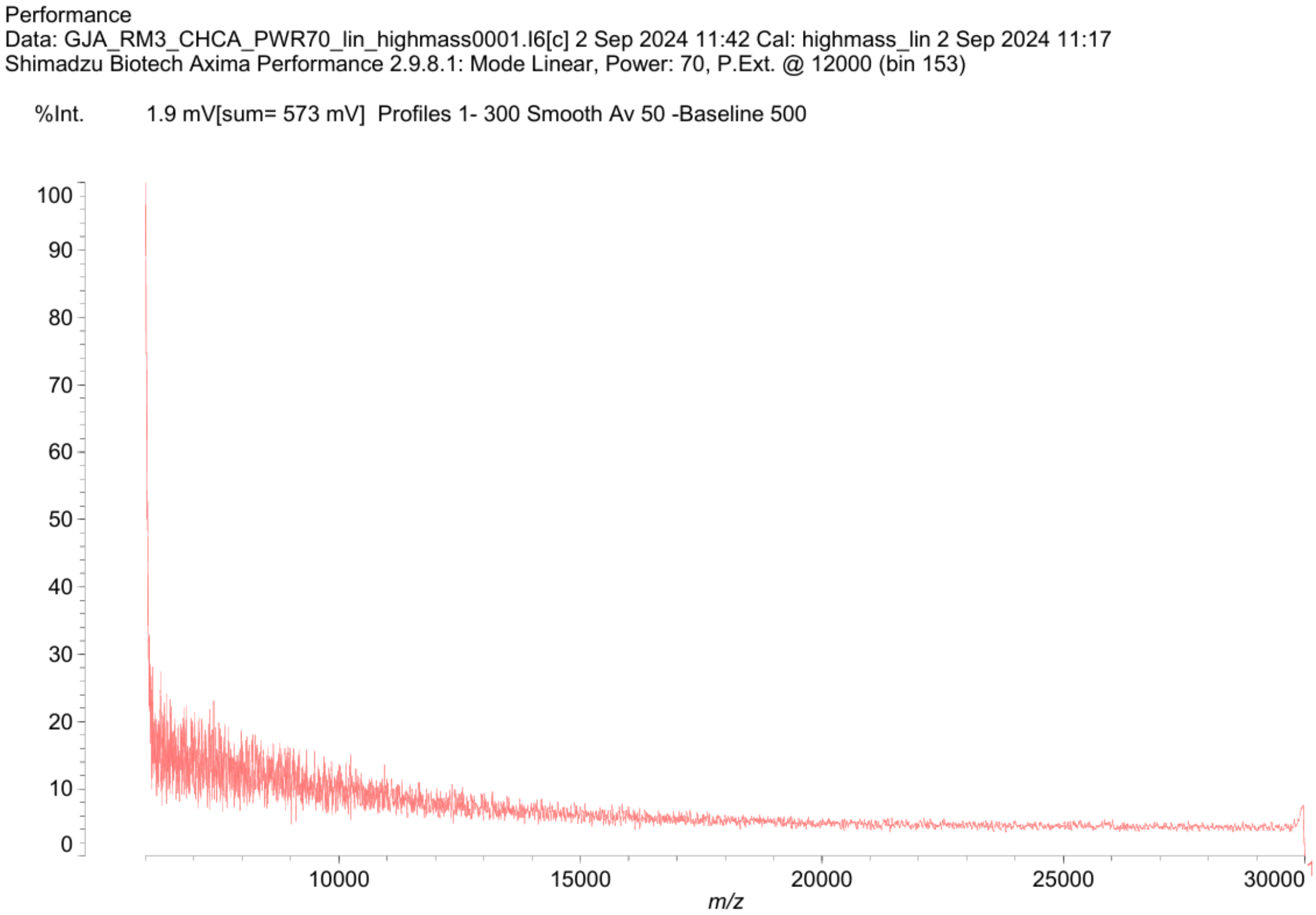

SI Figure 18. MALDI-MS of PEI 1.2 kDa (5000-30000 m/z).

3.5.2 10 kDa PEI

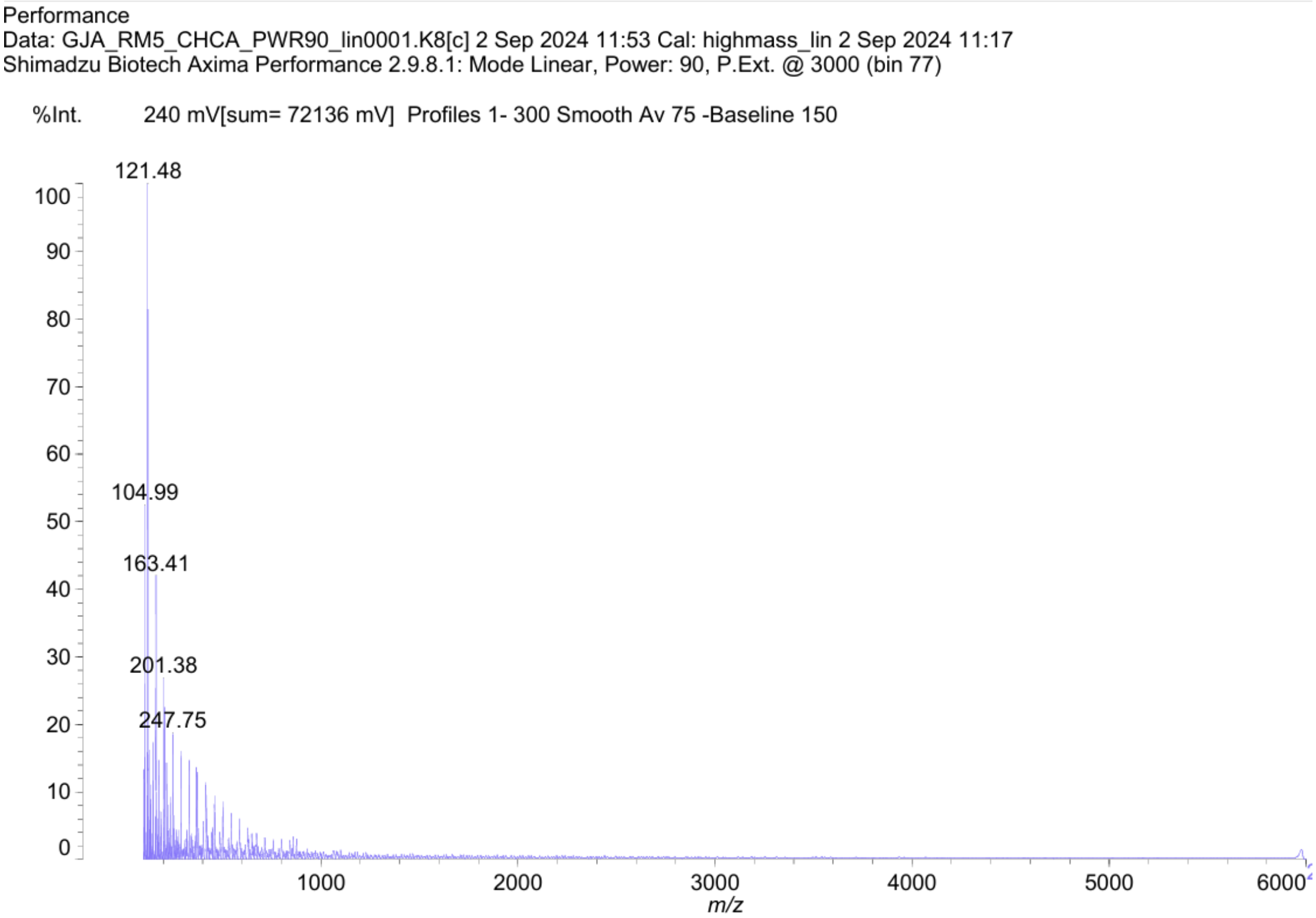

SI Figure 19. MALDI-MS of PEI 10 kDa (0-6000 m/z).

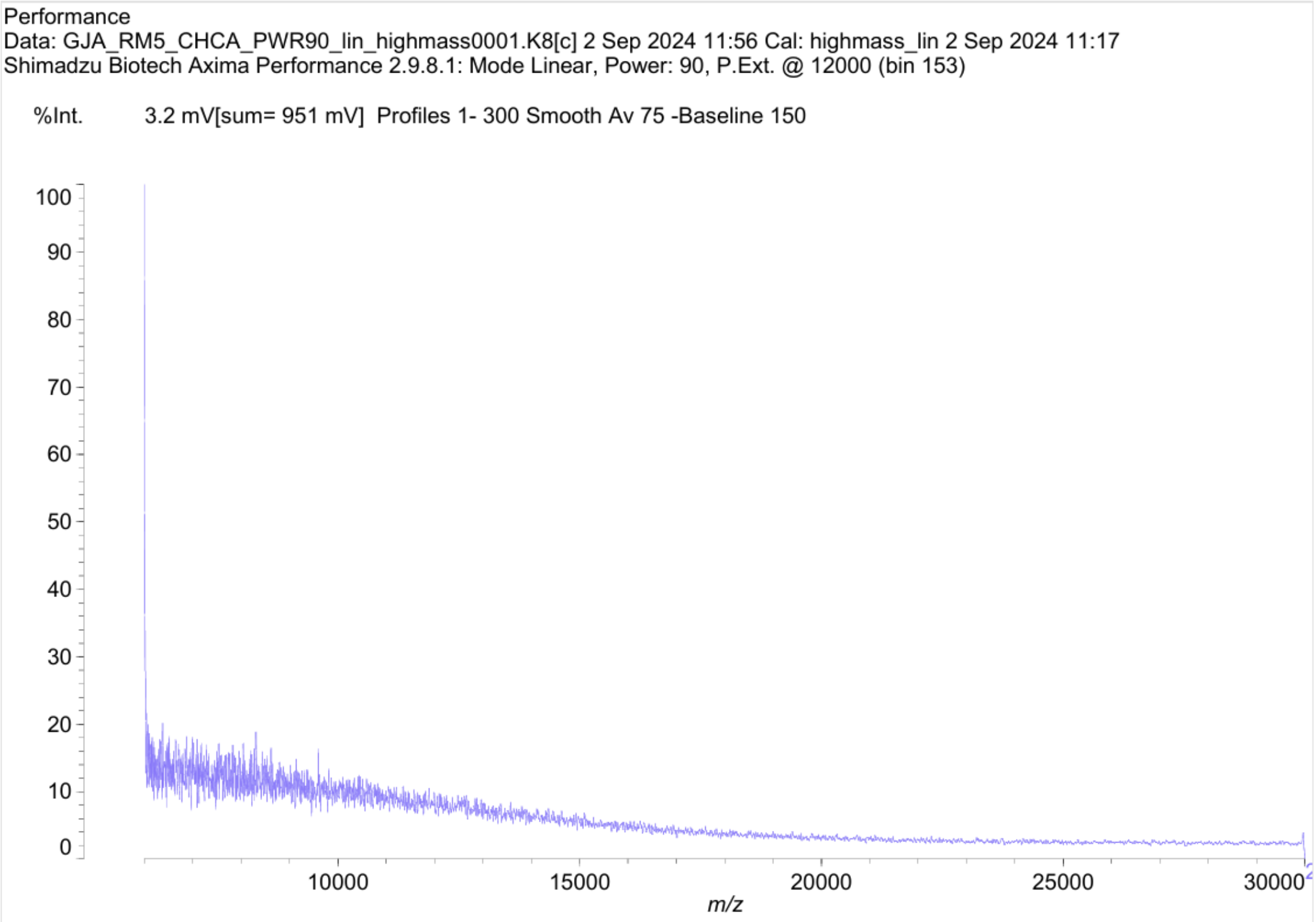

SI Figure 20. MALDI-MS of PEI 10 kDa (5000-30000 m/z).

3.5.3 25 kDa PEI

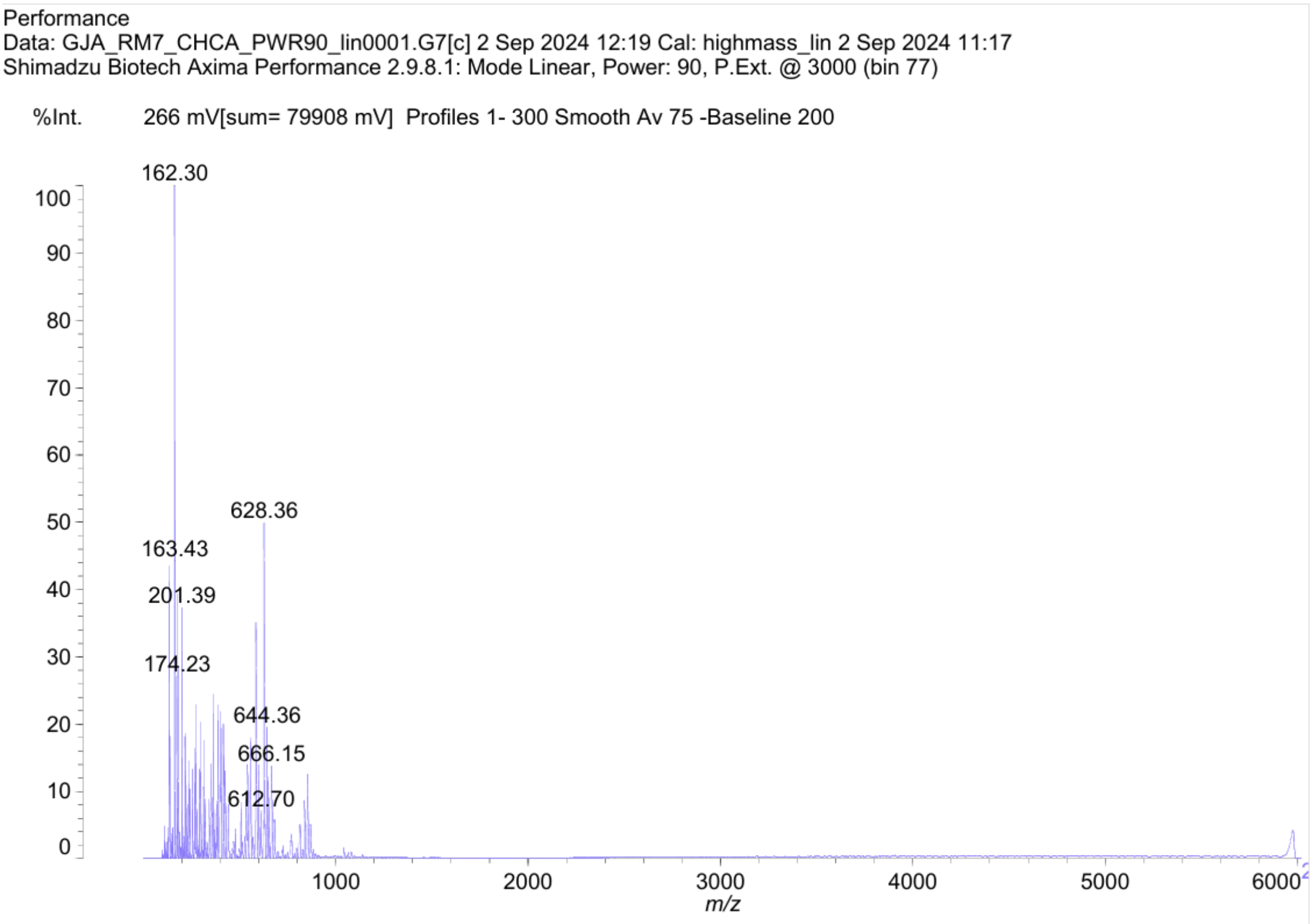

SI Figure 21. MALDI-MS of PEI 25 kDa (0-6000 m/z).

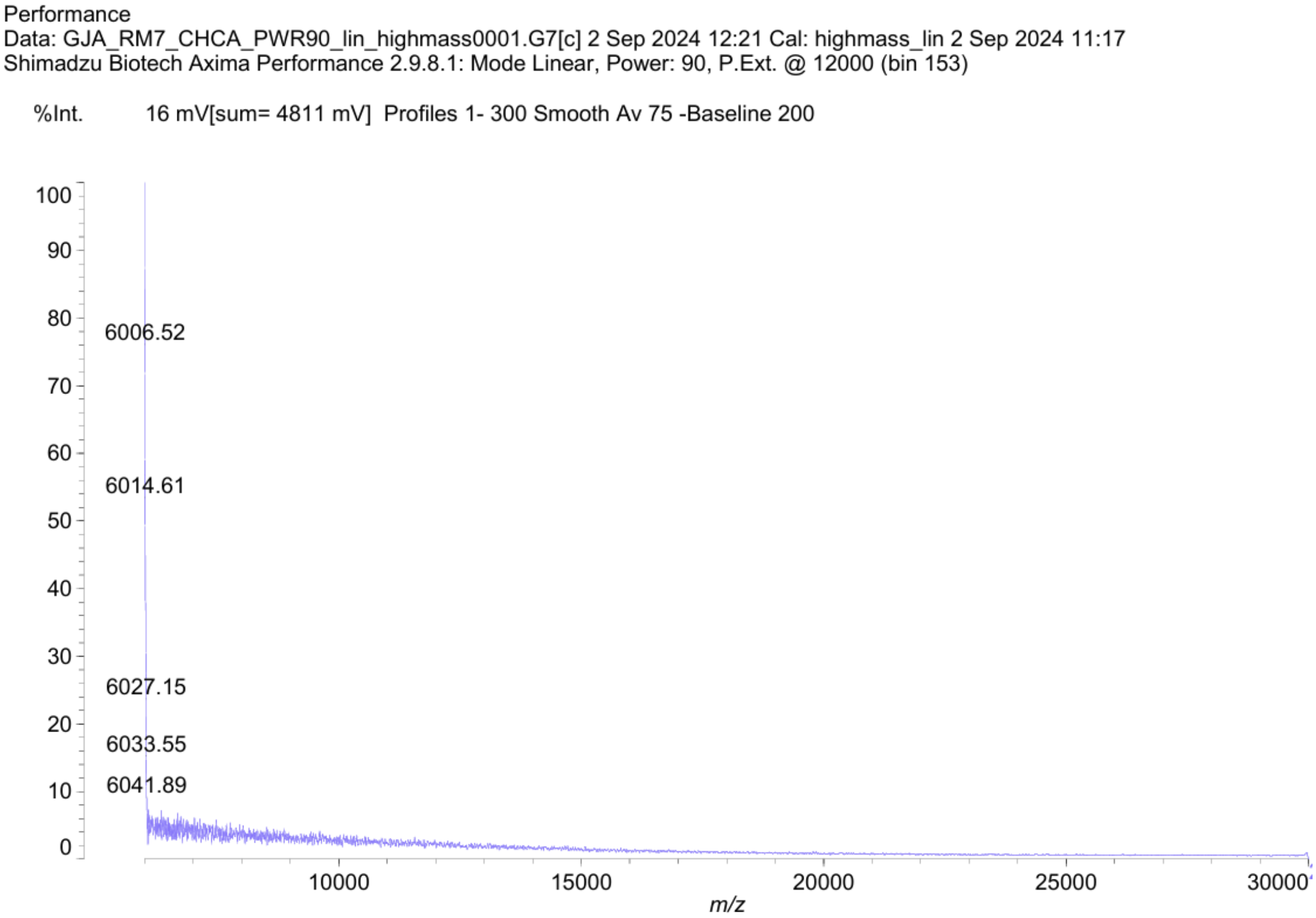

SI Figure 22. MALDI-MS of PEI 25 kDa (5000-30000 m/z).

Supplementary References

(1) *The fluorescence laboratory. - Calculate spectral overlap integral in FRET*. https://www.fluortools.com/software/ae-uv-vis-ir-spectral-software/ae-documentation-pages/aes-spectral-tools/calculate-spectral-overlap-integral-in-fret (accessed 2026-09-10)

(2) Shrestha, D.; Jenei, A.; Nagy, P.; Vereb, G.; Szöllősi, J. Understanding FRET as a Research Tool for Cellular Studies. *Int. J. Mol. Sci.* **2015**, *16* (4), 6718–6756. https://doi.org/10.3390/ijms16046718

(3) Ma, L.; Yang, F.; Zheng, J. Application of Fluorescence Resonance Energy Transfer in Protein Studies. *J. Mol. Struct.* **2014**, *1077*, 87–100. https://doi.org/10.1016/j.molstruc.2013.12.071
